# DeepLayer-ID: A Lightweight Multi-Domain Forensic Framework for Real-Time Deepfake Detection in Resource-Constrained UAV Sensor Platforms

**DOI:** 10.3390/s26092705

**Published:** 2026-04-27

**Authors:** Nayef H. Alshammari, Sami Aziz Alshammari

**Affiliations:** 1 Department of Computer Science, Faculty of Computers and Information Technology, University of Tabuk, Tabuk 47512, Saudi Arabia; nh.alshammari@ut.edu.sa; 2 Department of Information Technology, Faculty of Computing and Information Technology, Northern Border University, Rafha 73213, Saudi Arabia

**Keywords:** deepfake detection, UAV forensics, aerial surveillance, multi-channel feature decomposition, frequency-domain forensics, residual noise analysis, transformer fusion, real-time detection

## Abstract

Unmanned aerial vehicle (UAV) imaging systems are increasingly deployed in surveillance, infrastructure monitoring, and smart-city applications, where the integrity of captured visual data is critical. Recent advances in generative models enable highly realistic deepfake manipulations that can compromise aerial sensor streams, particularly under real-world degradations such as motion blur, sensor noise, and compression artifacts. This paper introduces DeepLayer-ID, a degradation-aware multi-domain forensic framework specifically designed for UAV sensing environments. The proposed architecture decomposes forensic evidence into complementary spatial, frequency, and residual domains. A discrete wavelet transform module captures sub-band energy inconsistencies, while high-pass residual filtering isolates sensor pattern anomalies. A lightweight transformer-based fusion mechanism adaptively integrates cross-domain representations to enhance robustness under heterogeneous acquisition conditions. To emulate operational UAV pipelines, we construct a balanced dataset of 1096 aerial frames derived from the VisDrone2019-DET validation subset, incorporating synthetic manipulations and physics-consistent degradations. The experimental results show that DeepLayer-ID achieves 97.8% accuracy and 0.991 AUC, outperforming ResNet-50 (90.9%, 0.942 AUC), XceptionNet (92.4%, 0.957 AUC), and Noiseprint CNN (93.1%, 0.964 AUC). Notably, the model maintains real-time feasibility, with only 5.4 M parameters and 9.8 ms inference latency. These findings demonstrate that structured multi-domain signal decomposition combined with attention-guided fusion provides a robust and computationally efficient solution for deepfake detection in degraded UAV sensing systems.

## 1. Introduction

The accelerated advancement of generative artificial intelligence has fundamentally reshaped the landscape of digital content authenticity, enabling the synthesis of visually indistinguishable deepfake imagery through diffusion models and adversarial generative networks [1]. These models learn high-dimensional data manifolds capable of reproducing fine-grained texture statistics, photometric consistency, and semantic coherence, thereby challenging traditional forensic assumptions regarding pixel-level irregularities [2]. While deepfake detection has predominantly focused on frontal facial datasets captured under controlled terrestrial conditions, the rapid proliferation of unmanned aerial vehicles (UAVs) in surveillance, urban intelligence, infrastructure monitoring, and biometric reconnaissance introduces a novel and underexplored threat surface [3]. UAV imaging systems operate as distributed sensing nodes within cyber-physical environments, where visual integrity directly influences autonomous perception, tracking, and decision-making pipelines [4]. The integration of generative manipulation techniques into aerial sensor streams permits adversaries to inject forged identities, modify scene topology, or fabricate dynamic agents within operational footage, thereby undermining trust in sensor-driven situational awareness and automated threat assessment [5].

Despite the rapid progress of image forgery detection, the forensic problem in UAV-based sensing environments remains fundamentally different from conventional deepfake analysis. In aerial surveillance, manipulated content is often embedded within wide-scene imagery, cluttered backgrounds, dynamic viewpoints, and small-scale objects, making forged regions substantially harder to isolate and verify. As a result, detection models designed for ground-level facial imagery or visually clean benchmarks often fail to generalize reliably when deployed in realistic UAV platforms. This mismatch motivates the need for a dedicated forensic framework that is not only manipulation-sensitive but also explicitly aware of the sensing constraints and degradation patterns that characterize aerial image acquisition.

Unlike ground-level imagery, UAV-acquired data exhibits intrinsic acquisition heterogeneity arising from platform dynamics and environmental variability [6]. Aerial perspectives introduce scale compression, multi-depth parallax, occlusion complexity, and rapid viewpoint shifts governed by pitch, roll, and yaw fluctuations. Moreover, UAV sensors function under constrained hardware and bandwidth conditions, resulting in motion blur, rolling-shutter distortions, stochastic sensor noise, atmospheric scattering, illumination inconsistency, and lossy JPEG compression [7]. These degradations alter both spatial and spectral signal distributions, attenuating conventional manipulation cues and complicating anomaly detection [8]. Contemporary generative models increasingly preserve global semantic realism while introducing subtle local inconsistencies in edge continuity, high-frequency sub-band energy, and sensor noise statistics [9]. However, under aerial degradations, such inconsistencies become statistically diluted, necessitating detection frameworks that explicitly model multi-domain signal behavior and remain invariant to operational sensing artifacts [10]. Therefore, deepfake detection in UAV environments must transition from purely appearance-based classification toward structured forensic decomposition grounded in signal theory and sensor-aware modeling [11].

To address these challenges, this work proposes DeepLayer-ID, a degradation-aware multi-domain forensic architecture specifically engineered for aerial sensing systems. The key innovation of the proposed framework lies in its ability to jointly analyze complementary forensic evidence across spatial, frequency, and residual domains rather than relying on a single-stream visual representation. This design is particularly important in UAV imagery, where manipulations may remain visually subtle in the RGB domain but still introduce detectable inconsistencies in high-frequency sub-bands or sensor noise residuals. Accordingly, the proposed framework is designed not only to improve detection accuracy but also to strengthen robustness under the degradations and operational uncertainties that define real-world aerial surveillance environments. The framework decomposes UAV imagery into complementary representational domains to expose generative inconsistencies that persist across acquisition distortions. First, a spatial RGB branch captures geometric boundary misalignments and texture discontinuities through lightweight convolutional encoding. Second, a discrete wavelet transform (DWT)-based frequency branch isolates directional high-frequency sub-bands (LH, HL, HH), amplifying spectral anomalies introduced by diffusion-based synthesis and blending operations. Third, a residual forensic branch leverages structured high-pass filtering to emphasize deviations in sensor pattern noise and compression-induced artifacts that generative models fail to faithfully replicate. These heterogeneous embeddings are subsequently aligned within a shared latent manifold and integrated using a multi-head transformer fusion module that enforces long-range relational consistency across scene entities. By coupling localized anomaly extraction with global semantic verification, DeepLayer-ID establishes a cross-domain anomaly consensus mechanism capable of discriminating manipulated content under complex UAV-induced degradations.

Beyond methodological innovation, the proposed framework is designed for operational feasibility within embedded aerial platforms. The architecture balances representational richness and computational efficiency through lightweight backbones and optimized attention mechanisms, enabling real-time inference compatible with onboard UAV processors. A physics-consistent preprocessing pipeline further enhances robustness by modeling motion blur, stochastic sensor noise, compression artifacts, and illumination variability, reflective of real-world flight conditions. Through this sensor-aligned and degradation-resilient design, DeepLayer-ID advances deepfake forensics from static image classification toward resilient cyber-physical sensing security, reinforcing trust in autonomous aerial monitoring, biometric authentication, and intelligent surveillance infrastructures.

The main contributions of this work are summarized as follows:

- We formulate deepfake detection in UAV imagery as a degradation-aware aerial forensic problem, explicitly accounting for the distinctive challenges of aerial sensing, including small manipulated regions, scene clutter, viewpoint variation, motion blur, sensor noise, and compression artifacts.
- We propose *DeepLayer-ID*, a lightweight multi-domain forensic architecture that jointly exploits spatial, frequency, and residual representations together with transformer-based cross-domain fusion, to capture complementary manipulation cues in degraded UAV imagery.
- We establish an operationally grounded UAV forensic pipeline by combining a balanced VisDrone-based deepfake benchmark with physics-consistent degradation modeling, thereby supporting robust and deployment-oriented evaluation for real-time embedded UAV surveillance environments.

**Figure 1:**
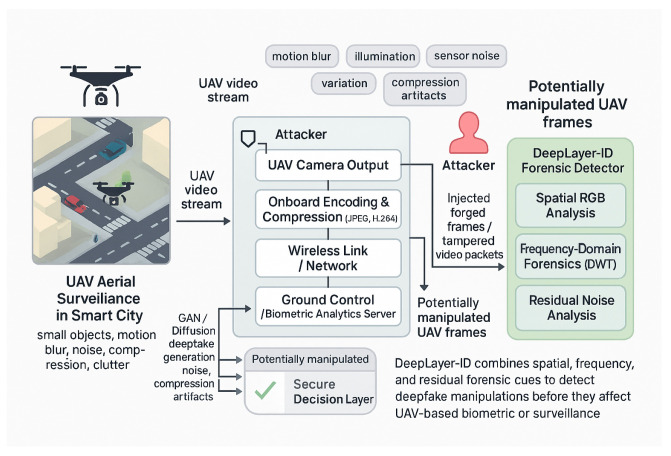
UAV deepfake threat and DeepLayer-ID protection overview. A UAV captures complex aerial scenes in a smart-city environment.

This study therefore directly contributes toward securing UAV-based biometric authentication and aerial situational awareness against emerging deepfake threats. Figure 1 illustrates the UAV deepfake threat scenario and the role of the proposed DeepLayer-ID framework within the aerial surveillance pipeline.

## 2. Literature Review

Tanfoni (2025) [12] proposed an explainable deepfake detection framework that integrates facial segmentation with SHAP-based interpretability to mitigate the black-box behavior of convolutional neural networks. Their study addresses the problem of CNN overfitting to highly salient facial regions while neglecting contextual cues, which reduces generalization and interpretability. Their methodology combines DeepLabV3+ for face–background separation with transfer-learned CNN backbones, followed by SHAP attribution analysis to visualize decision-driving regions. Experimental results demonstrate improved classification reliability and transparency compared to conventional CNN baselines, confirming that segmentation-guided attention enhances interpretability. However, their work is evaluated primarily on near-frontal facial datasets under controlled imaging conditions and does not report robustness under motion blur, scale reduction, or aerial distortions. Furthermore, it lacks multi-domain forensic decomposition (spatial–frequency–residual). In contrast, our DeepLayer-ID framework explicitly addresses UAV-specific degradations and integrates cross-domain feature fusion to ensure robust detection in aerial biometric surveillance environments.

Fouad et al. (2025) [13] investigated double JPEG compression detection by modeling cross-band DCT frequency correlations in a multi-branch convolutional architecture. Their research problem focuses on identifying recompression artifacts under unknown quantization tables, a common forensic challenge in image tampering. Their approach extracts inter- and intra-band DCT correlations and incorporates quantization metadata to improve robustness. Evaluated on the large-scale Park dataset containing over one million manipulated patches, the method achieved 94.15% classification accuracy, outperforming VGG16, DenseNet121, and ResNet50 baselines while maintaining approximately 5.95 M parameters. Although highly effective for compression-based manipulations, their method is limited to recompression artifacts and does not address semantic deepfakes generated by GANs or diffusion models. It also does not consider UAV-induced degradations such as motion blur, altitude-based scale compression, or sensor noise. Our DeepLayer-ID model extends beyond compression forensics by integrating spatial, wavelet-based frequency, as well as residual-noise cues specifically tailored to aerial manipulation scenarios.

Hamdi (2021) [14] explored unsupervised anomaly detection in UAV surveillance videos to mitigate operator overload and the scarcity of labeled abnormal events. The problem addressed is the detection of unusual crowd or motion behavior in aerial streams without supervised anomaly labels. Their methodology combines VGG16 spatial descriptors, optical-flow histograms, One-Class SVM classifiers, and convolutional autoencoders for motion-based anomaly modeling. Their study reported an AUC of 85.3% in aerial anomaly detection experiments, demonstrating reliable abnormality discrimination under real UAV conditions. However, their system targets behavioral anomalies rather than adversarial image manipulation or biometric spoofing. It does not model synthetic identity swaps, object insertion artifacts, or generative texture inconsistencies. Our DeepLayer-ID framework directly addresses manipulation-level forensics by learning multi-domain generative artifact representations rather than motion irregularities alone.

Tiwari (2023) [15] provided a comprehensive survey of spoofing threats across biometric modalities, including face, iris, and fingerprint systems. Their study addresses vulnerabilities to presentation attacks such as printed images, replay attacks, and 3D masks, emphasizing the need for liveness detection and multimodal defense strategies. Their methodology synthesizes existing machine learning-based anti-spoofing approaches and categorizes biometric threat models. While their survey highlights critical weaknesses in single-modality defenses, it remains conceptual and does not propose or empirically validate a UAV-deployable spoof detection architecture. It does not consider small-scale biometric targets, aerial blur, or environmental noise. Our DeepLayer-ID framework operationalizes spoof-resistant detection under UAV constraints by combining spatial boundary modeling, frequency analysis, and residual forensic cues.

Chakraborty et al. (2025) [16] introduced TruthLens, a training-free deepfake authentication framework that reframes detection as a Visual Question Answering (VQA) task using large vision–language models and GPT-based reasoning. The problem addressed is the lack of interpretability and generalization in conventional binary CNN classifiers. Their methodology employs LVLM probing with semantic reasoning prompts to produce interpretable authenticity verdicts. Their model achieved AUC values of 95.0% on LDM-generated images and 97.5% on ProGAN data, significantly outperforming CNNDetection and DIRE baselines. Despite strong interpretability and cross-model generalization, this approach requires heavy computational resources due to large language–vision inference, limiting its suitability for real-time embedded UAV systems. It also lacks explicit modeling of aerial distortions and multi-channel forensic evidence. Our DeepLayer-ID architecture achieves real-time efficiency (9.8 ms inference, 5.4 M parameters) while integrating spatial, spectral, and residual cues specifically designed for UAV surveillance.

Bartusiak and Delp (2022) [17] investigated synthetic audio deepfake detection by transforming speech signals into spectrogram images and applying CNN-based classification. The problem addressed is the identification of neural speech synthesis artifacts in the frequency domain. Their methodology relies on spectrogram feature extraction and convolutional discrimination across ASVspoof2019 benchmarks. Their model achieved 85.99% accuracy, with an ROC-AUC of 0.9012, demonstrating strong frequency-domain discriminative power. However, this approach is limited to the audio modality and does not generalize to visual aerial forgeries. It does not integrate the spatial boundary or residual noise modeling required for image-based spoof detection. Inspired by the effectiveness of spectral cues, our DeepLayer-ID framework incorporates DWT-based frequency decomposition within a multi-domain visual forensic architecture.

Khan et al. (2025) [18] conducted a systematic review of generative AI-based synthetic media detection methods, addressing the problem of limited cross-domain robustness and poor real-world generalization. Their survey analyzes supervised, self-supervised, explainable, and lightweight architectures, highlighting the importance of hybrid forensic strategies. While their study does not present a single benchmark metric, it identifies multi-domain feature integration and efficiency-aware modeling as key future research directions. However, it does not propose or validate a UAV-specific implementation under aerial distortions. Our DeepLayer-ID framework directly responds to these recommendations by combining spatial, frequency, and residual features within a lightweight transformer-fusion architecture optimized for UAV deployment.

Gupta et al. (2025) [19] introduced MultiFakeVerse, a large-scale dataset targeting scene-level and person-centric semantic deepfake manipulations. The problem addressed is the detection of subtle contextual edits beyond traditional face swaps. The dataset contains 845,286 images, and evaluation results show PSNR = 66.30 dB, SSIM = 0.5774, and FID = 3.30, indicating high realism of generated content. Human evaluation achieved only 61.67% classification accuracy, and zero-shot detector performance dropped to 66.87% in overall accuracy, highlighting the difficulty of semantic forgeries. Despite advancing dataset diversity, the benchmark does not consider UAV viewpoints, small-scale targets, or sensor-originated distortions common in aerial capture. Our DeepLayer-ID model addresses these limitations by explicitly modeling geometric consistency, spectral anomalies, and residual sensor inconsistencies in UAV imagery.

Shao et al. (2025) [20] proposed MGMA-DSCNN, a lightweight tampering detection model integrated into an edge–cloud IoMT framework. The problem addressed is achieving high tampering detection accuracy under hardware constraints. Their methodology combines multi-scale gated attention with depth-wise separable convolutions to reduce computational overhead. Experimental evaluation reported 98.1% classification accuracy with low latency, validating its efficiency. However, the system targets general multimedia streams rather than aerial still-image deepfakes and does not incorporate explicit frequency-domain decomposition or residual forensic modeling tailored to UAV distortions. Our DeepLayer-ID framework maintains comparable efficiency while explicitly addressing aerial manipulation artifacts through multi-channel decomposition and transformer-based semantic fusion.

## 3. Methodology

The proposed methodology, illustrated in a DeepLayer-ID flowchart, establishes a structured and UAV-aware forensic pipeline that systematically transforms raw aerial imagery into a manipulation-aware decision framework. It is important to note that the proposed framework is designed for single-frame UAV image analysis and does not incorporate temporal modeling across consecutive video frames. The process begins with a balanced dataset construction, where authentic VisDrone UAV frames are paired with synthetically generated deepfake counterparts to ensure statistical symmetry and manipulation diversity. The pipeline is then processed through an degradation-consistent preprocessing module specifically engineered to emulate operational UAV imaging conditions, including aspect-ratio-preserving resizing, motion blur simulation, adaptive sensor noise modeling, JPEG compression emulation, and CLAHE-based illumination normalization. Importantly, conditional validation stages are embedded within the flow to ensure preprocessing integrity and prevent distortion-induced forensic bias. The standardized frames are subsequently dispatched into a multi-branch DeepLayer-ID architecture, where spatial RGB features, DWT-based frequency components, and residual forensic cues are extracted in parallel using lightweight encoders. These heterogeneous feature representations are aligned and fused via a transformer-based cross-branch attention mechanism that enforces global semantic coherence and long-range contextual reasoning before producing a final real/fake classification. The model focuses on spatial and intra-frame forensic inconsistencies, whereas temporal artifacts such as inter-frame flickering, motion continuity anomalies, and cross-frame semantic inconsistency remain beyond the current scope of this study. The inclusion of decision nodes within the workflow emphasizes validation checkpoints and model-driven inference control, reinforcing robustness against superficial artifacts. Collectively, the flowchart depicts a deeply integrated forensic system that unifies degradation modeling, multi-domain feature decomposition, and transformer-guided fusion to achieve reliable, real-time deepfake detection in complex UAV surveillance environments.

**Figure 2:**
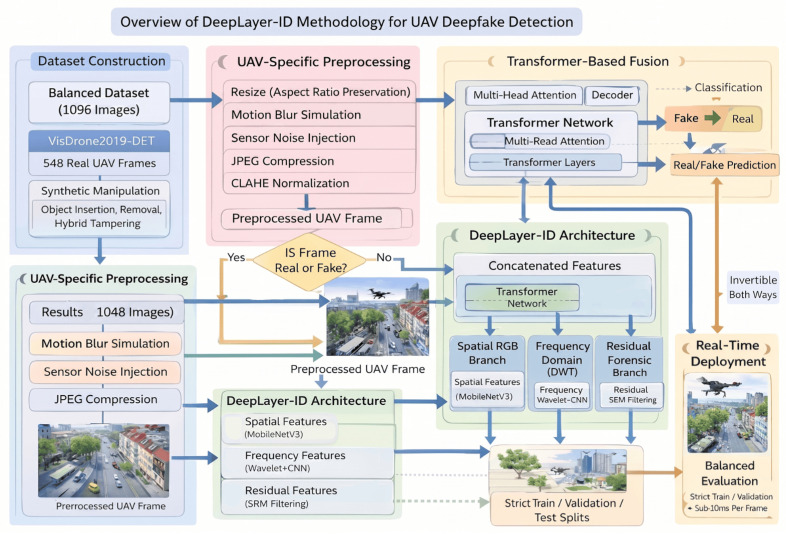
Step-by-step workflow of the proposed DeepLayer-ID framework. An input UAV frame from the balanced authentic/manipulated dataset is first preprocessed using UAV-specific degradation modeling, then passed through spatial, frequency, and residual forensic branches, followed by transformer-based fusion and final real/fake classification.

Figure 2 can be interpreted as a sequential data flow pipeline. First, an input UAV frame is selected from the balanced dataset, which contains both authentic VisDrone images and their synthetically manipulated counterparts. Second, the selected frame is passed through the UAV-specific preprocessing module, where aspect-ratio-preserving resizing, motion blur simulation, sensor noise injection, JPEG compression emulation, and CLAHE-based illumination normalization are applied to generate a standardized and degradation-aware input representation. Third, the preprocessed UAV frame is forwarded to the DeepLayer-ID architecture, where it is decomposed into three parallel forensic streams: a spatial RGB branch for texture and boundary analysis, a DWT-based frequency branch for high-frequency artifact extraction, and a residual forensic branch for sensor noise and compression trace analysis. Fourth, the feature embeddings produced by these three branches are aggregated and passed to the transformer-based fusion module, which performs cross-branch attention and contextual refinement. Finally, the fused representation is processed by the classifier to produce the real/fake prediction, which is then used for model training, validation, testing, or real-time deployment.

### 3.1. Dataset Description

This study employs a UAV image dataset constructed from the VisDrone2019-DET validation subset and its corresponding synthetically manipulated deepfake counterparts. The authentic portion of the dataset consists of real aerial images captured under diverse urban surveillance conditions, while the manipulated portion was generated to emulate realistic deepfake attacks relevant to UAV-based monitoring environments. Specifically, the dataset is designed to support the training and evaluation of the proposed DeepLayer-ID forensic framework under realistic aerial imaging conditions, including variations in scale, viewpoint, illumination, motion blur, and scene complexity.

The use of the VisDrone2019-DET validation subset is appropriate for this study because it provides high-resolution UAV imagery captured across a wide range of real-world outdoor scenes, including roads, intersections, pedestrian areas, commercial environments, residential blocks, and open urban spaces. These characteristics make the dataset well suited for investigating deepfake detection in UAV surveillance scenarios, where manipulated regions may appear at different spatial scales and under heterogeneous environmental conditions.

To enable supervised forensic learning, the authentic UAV frames were paired with synthetically generated manipulated samples created through scene-level editing, identity-level facial manipulation, and hybrid composite alterations. As a result, the final dataset supports binary classification between authentic and manipulated UAV imagery while preserving the operational realism required for robust forensic evaluation.

### 3.2. Dataset Characteristics and Metadata

The VisDrone2019-DET validation subset provides not only high-resolution UAV imagery but also an extensive collection of annotations describing object categories, scene composition, and contextual attributes. Although these annotations are not directly used as supervisory labels for the proposed forensic detector, they offer valuable semantic information that helps characterize the structural and visual complexity of the dataset. The inherent diversity of VisDrone imagery, combined with its rich annotation set, creates a challenging environment for detecting deepfake manipulations, particularly when adversaries attempt to alter object appearance, insert synthetic agents, or modify scene topology.

The dataset includes a wide range of geographic and environmental settings, such as straight urban road segments, busy intersections, public areas with large numbers of pedestrians, commercial zones, residential blocks, and semi-structured open spaces. This variation captures the natural diversity encountered in real-world UAV deployments. Forensic detection models must therefore generalize across different backgrounds, where manipulation artifacts may be subtle and partially concealed by scene clutter.

#### 3.2.1. Camera Geometry and Perspective Variation

The imagery exhibits substantial variation in pitch, roll, and yaw due to the dynamic nature of UAV flight. These variations produce top-down, semi-vertical, and oblique viewing angles, introducing perspective distortion and scale inconsistency, which synthetic manipulation methods often struggle to replicate accurately. Deepfake detection systems must therefore identify inconsistencies in object geometry, shadow direction, and depth-related cues, all of which may indicate manipulated content.

#### 3.2.2. Illumination Variability and Environmental Lighting

The lighting conditions in the dataset vary considerably, ranging from direct overhead sunlight and deep shadows cast by buildings or trees to overcast scenes, dusk-level illumination, and glare from reflective surfaces such as vehicles or metallic objects. These lighting variations affect color distribution, shadow formation, and local contrast. Generative deepfake models often fail to reproduce such intricate illumination patterns accurately, making photometric inconsistency an important forensic indicator.

#### 3.2.3. Motion, Blur, and UAV-Induced Distortion

Mechanical vibration and rapid movement of UAV platforms introduce directional motion blur, atmospheric distortion, and rolling-shutter effects. Generative models often find it difficult to reproduce how these distortions interact with object boundaries, fine textures, and scene edges. The discrepancy between genuine UAV motion characteristics and synthetically generated content therefore serves as an important forensic cue exploited by the proposed multi-domain framework.

#### 3.2.4. Scene Complexity and Object Density

VisDrone scenes often contain a high object density, including vehicles, pedestrians, bicycles, tricycles, and other small-scale objects distributed across multiple depth planes. This density increases the likelihood of occlusion, overlap, and fine-grained interaction between scene elements. Deepfake manipulations must therefore preserve spatial relationships, scale consistency, and occlusion patterns to remain convincing. Any misalignment or geometric irregularity introduced during synthetic editing may be revealed through structural and semantic consistency analysis. To illustrate the diversity of objects present in the dataset, Table 1 provides representative examples of the annotated categories included in VisDrone. These categories highlight the semantic richness of the dataset and justify its suitability for evaluating forensic detection systems designed for UAV imagery.

### 3.3. Synthetic Deepfake UAV Frame Construction and Quality Control

Since the VisDrone2019-DET validation subset contains only authentic aerial imagery, it was necessary to construct a balanced and representative dataset of manipulated samples to enable supervised training of the proposed DeepLayer-ID forensic detection framework. To this end, a diverse collection of synthetic deepfake UAV frames was generated using a combination of state-of-the-art generative adversarial networks, diffusion-based inpainting models, and identity-level facial manipulation techniques. These methods collectively emulate realistic adversarial threats encountered in UAV surveillance, intelligence gathering, and remote monitoring systems. The resulting manipulations vary in semantic complexity, spatial scale, and structural coherence, thereby ensuring that the proposed model learns to detect a wide spectrum of potential forgery patterns.

Concerning the generation of the manipulated samples, a dedicated quality control stage was incorporated to ensure that the synthetic deepfake frames were visually plausible and not trivially distinguishable due to poor synthesis quality. Specifically, the generated manipulations were examined with respect to boundary continuity, texture consistency, illumination plausibility, shadow coherence, local geometric realism, and contextual blending with surrounding scene content. Samples exhibiting obvious synthesis failures, unrealistic color transitions, severe structural distortion, or visually implausible artifacts were excluded from the final dataset. This step was important to ensure that the final manipulated corpus represented challenging and realistic UAV adversarial forgeries rather than low-quality synthetic negatives that could artificially inflate forensic detection performance.

A. GAN-Based Scene Manipulation

High-fidelity generative adversarial networks (GANs) and diffusion models [21] were utilized to perform scene-level manipulations while preserving global appearance consistency. These models introduce forged scene elements or remove authentic ones through texture-aware editing. Specifically, the manipulation pipeline includes the following:

- Object insertion: Synthetic vehicles, pedestrians, bicycles, or structural objects are generated and blended into the scene with diffusion-guided edge harmonization, ensuring plausible shadowing and illumination.
- Object removal: Existing objects are removed and the underlying region is reconstructed using context-aware inpainting, which often leaves faint but detectable inconsistencies in texture continuity and local pixel gradients.
- Scene restructuring: Background elements such as building façades, roadside barriers, vegetation, and ground textures are modified to alter structural integrity without disrupting global scene context [22].

To maintain the visual quality of GAN- and diffusion-based scene edits, each generated sample was additionally screened for realistic object scaling, edge smoothness, photometric compatibility with the surrounding region, and consistency of inserted or reconstructed content with the original UAV viewpoint. This prevents the inclusion of manipulated samples with overly obvious artifacts unrelated to the actual forensic objective of this study.

B. Semantic Identity Deepfake Manipulation

For UAV frames containing human subjects, identity-level deepfake manipulations were introduced using high-resolution face-swapping models [23]. These methods replace the facial identity of individuals while preserving head pose, lighting direction, and environmental context. Such manipulations simulate adversarial impersonation scenarios frequently discussed in security and forensic applications, including law enforcement evasion or intentional misrepresentation of individuals during aerial reconnaissance. Although these regions occupy small spatial areas in UAV imagery, identity-level forgeries often introduce subtle inconsistencies in color distribution, boundary smoothness, and shadow coherence that DeepLayer-ID is designed to detect.

Because facial regions in UAV imagery are typically small and frequently affected by altitude, motion blur, and scale reduction, the quality control process for identity manipulations is particularly focused on preserving plausible facial blending, local illumination consistency, and boundary realism around the manipulated region. Samples with clearly unrealistic face transitions or visually unstable synthetic identity overlays were removed to ensure that the retained manipulations remained challenging and representative of realistic UAV-based identity forgery attempts.

C. Composite Overlays and Hybrid Manipulations

Isolated manipulation techniques and hybrid editing pipelines were applied to create composite deepfakes that combine multiple forgery strategies. These include the following:

- Local region tampering across multiple semantic layers;
- Shadow inconsistencies arising from incorrect lighting synthesis;
- Structural blending mismatches along object boundaries;
- Texture hallucination and pattern repetition within synthesized regions.

These hybrid manipulations substantially increase the diversity and complexity of the synthetic dataset, exposing DeepLayer-ID to a broad range of artifacts that may occur in operational UAV forgeries. Since hybrid manipulations are more likely to accumulate multiple visual inconsistencies, they were also reviewed for overall scene plausibility so that unrealistic combinations of artifacts were not over-represented in the final dataset.

All synthetic frames underwent the same preprocessing pipeline applied to the real VisDrone images, including resizing, normalization, blur modeling, noise injection, compression simulation, and illumination normalization. This ensures dataset homogeneity and prevents statistical separation between authentic and manipulated samples based solely on preprocessing inconsistencies.

To further support the realism of the generated corpus, the quality assessment was not treated as a separate classification label but as a filtering mechanism during dataset construction. In other words, the synthetic frames were retained only when the manipulations preserved sufficient perceptual plausibility to resemble realistic adversarial edits under UAV imaging conditions. This is important because the objective of the proposed framework is to detect subtle and operationally meaningful forgeries rather than visually obvious synthetic artifacts.

**Figure 3:**
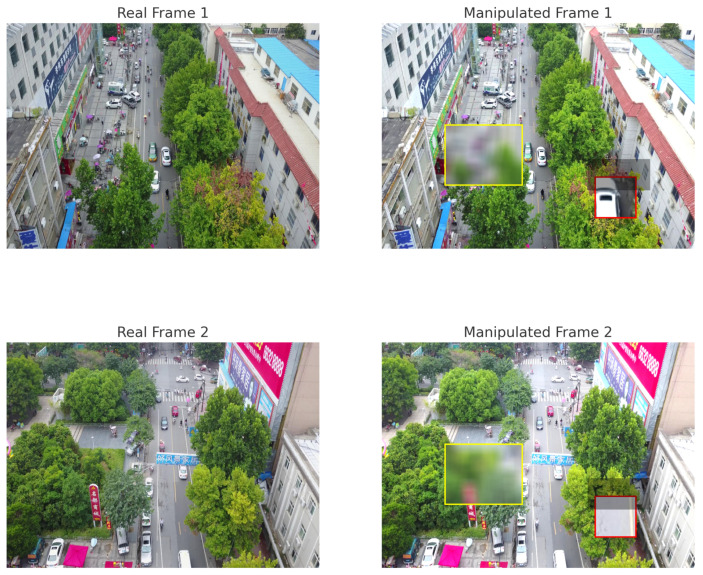
Examples of authentic VisDrone UAV frames and their manipulated counterparts, illustrating scene-level forgeries such as object removal, object insertion, illumination inconsistencies, and shadow mismatches. The shown manipulated samples are representative examples from the quality-controlled synthetic dataset used in this study.

To illustrate the differences between authentic UAV imagery and the corresponding synthetic manipulations generated by the proposed pipeline, Figure 3 presents multiple examples of real VisDrone frames alongside their manipulated counterparts. The synthetic forgeries shown in this figure incorporate several controlled yet realistic tampering operations, such as object removal through texture-based inpainting, object insertion via composite blending, illumination inconsistencies, and shadow mismatches, which mirror the types of adversarial edits expected in real UAV surveillance attacks. Each of these modifications introduces a distinct category of scene-level or physical inconsistency: inpainting alters structural continuity and background geometry, inserted objects disrupt semantic and spatial coherence, and lighting and shadow distortions violate photometric realism. These targeted distortions are crucial to the objectives of this study, as they expose the types of multi-layer anomalies that DeepLayer-ID is designed to detect. By presenting several varied and intentionally challenging examples, this figure highlights how the synthetic dataset stresses the model across geometric, textural, semantic, and illumination dimensions, while also illustrating the visually plausible quality of the retained manipulated samples after the quality control process.

#### Train/Validation/Test Split

To ensure a rigorous and unbiased evaluation of the proposed DeepLayer-ID framework, the combined dataset consisting of 548 original VisDrone2019-DET-val UAV images and an equal number of synthetically generated deepfake frames was partitioned into three non-overlapping subsets for training, validation, and testing. This partitioning strategy was designed to preserve the statistical diversity of the dataset while preventing cross-contamination between the authentic and the manipulated samples across different phases of model development.

A stratified sampling approach was employed to guarantee that each subset contained equal proportions of real and synthetic images, as well as a balanced distribution of distinct manipulation categories. This ensures that the model does not acquire biases related to sample ordering, manipulation density, or contextual scene types. The partitioning scheme follows a standard 70/15/15 ratio, optimizing both learning capacity and evaluation reliability.

- Training Set (70%)-the largest portion of the dataset, used to train the multilayer decomposition module, multi-branch feature extraction components, and the cross-layer fusion mechanism. This subset includes a balanced representation of authentic and manipulated UAV frames to promote robust forensic learning across heterogeneous visual conditions.
- Validation Set (15%)-a separate group used only for optimizing hyperparameters, calibrating fusion weights, and setting early stopping criteria. The validation samples enable the model to generalize to unobserved manipulations, ensuring that the training process does not overfit particular forgery patterns or scene structures.
- Test Set (15%)-a set of real UAV images and synthetically altered frames that are not used during training or validation. This subset offers an impartial standard for evaluating the model’s ultimate performance in authentic UAV forensic scenarios.

To avoid ambiguity in the experimental workflow, the input preparation strategy was explicitly divided into two categories: universal preprocessing and training-only degradation-aware augmentation. Universal preprocessing operations, namely aspect-ratio-preserving resizing and pixel normalization, were applied consistently to the training, validation, and test sets in order to ensure dimensional and statistical consistency across all samples. In contrast, UAV-specific degradation simulation operations, including motion blur simulation, sensor noise injection, JPEG compression emulation, and illumination normalization, were applied only to the training set as robustness-oriented augmentation. These degradation operations were not applied to the validation or test sets, thereby preserving the integrity of the held-out evaluation protocol and ensuring that performance improvements reflect generalization rather than artificial exposure to augmented variants. Table 2 summarizes the complete composition of the combined dataset and its distribution across the three evaluation subsets, while the split-specific preprocessing operations are clarified in the corresponding preprocessing description.

Although the total dataset size of 1096 images is smaller than that of large-scale generic computer vision benchmarks, its use in this study is justified by the domain-specific nature of UAV forensic analysis and the design of the proposed framework. The dataset is strictly balanced, containing 548 authentic UAV frames and 548 manipulated counterparts, and is partitioned using a stratified 70%/15%/15% protocol to preserve class balance and manipulation diversity across training, validation, and testing subsets. In addition, the proposed DeepLayer-ID architecture is intentionally lightweight rather than excessively parameterized, making it more suitable for learning from a curated and degradation-aware UAV dataset than from a massive unconstrained corpus. The preprocessing pipeline further strengthens the effective training value of the dataset by exposing the model to realistic UAV degradations, including motion blur, sensor noise, JPEG compression, and illumination variation. Therefore, the dataset is designed to prioritize forensic relevance, class balance, and operational realism rather than scale alone. Nevertheless, we acknowledge that future work should validate the framework on larger and more diverse UAV deepfake datasets to further assess generalization.

### 3.4. Data Preprocessing Pipeline

A preprocessing pipeline serves as a critical foundational stage of the DeepLayer-ID framework, ensuring that all UAV imagery, whether real or synthetically manipulated, undergoes a consistent series of operations prior to multilayer decomposition and neural feature extraction. UAV-captured frames are often characterized by non-uniform resolutions, dynamic motion distortion, atmospheric interference, sensor noise, and heterogeneous illumination conditions. Such factors make raw imagery unsuitable for direct ingestion by a forensic detection model, particularly one that is sensitive to subtle manipulation artifacts. To ensure methodological clarity and consistency across dataset splits, the input preparation strategy in this study is explicitly divided into two components: (i) universal preprocessing operations applied to all samples, and (ii) UAV-specific degradation simulation applied only to the training set as robustness-oriented augmentation. Thus, the preprocessing pipeline fulfills two objectives:

1. Normalizing real and synthetic images into a unified feature space;
2. Simulating UAV-specific degradations that strengthen the model’s generalization to real operational environments during training only.

More specifically, aspect-ratio-preserving resizing and pixel normalization are treated as core preprocessing operations and are applied consistently to the training, validation, and test sets. In contrast, motion blur simulation, sensor noise injection, JPEG compression emulation, and illumination normalization via CLAHE are used only for training samples to expose the model to realistic UAV acquisition artifacts and improve robustness under degraded sensing conditions. These augmentation-oriented degradation steps are not applied to the validation or test sets, thereby preserving the integrity of the held-out evaluation protocol and avoiding ambiguity in the experimental workflow.

**Figure 4:**
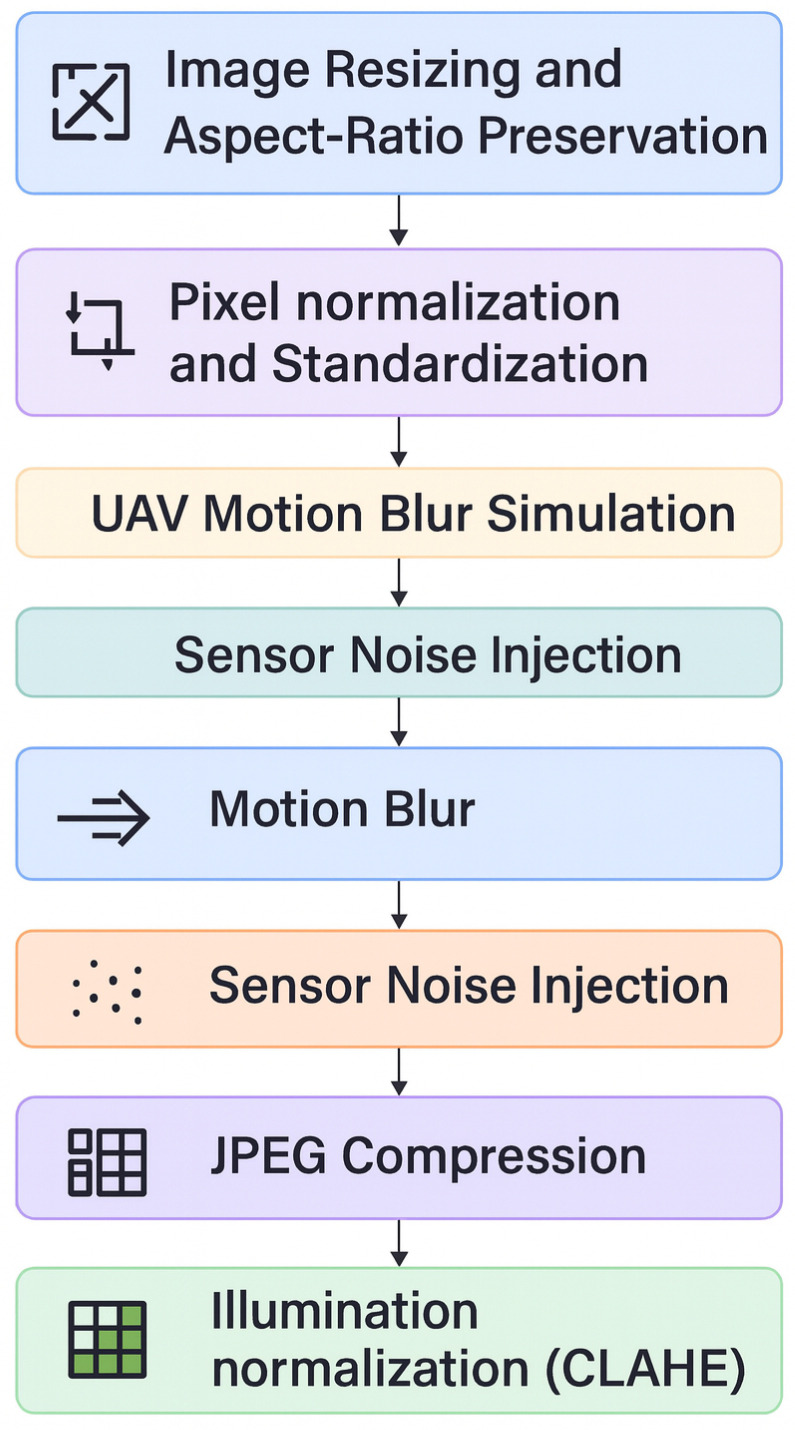
Overview of the DeepLayer-ID input preparation pipeline. Core preprocessing operations, including aspect-ratio-preserving resizing and pixel normalization, are applied to all UAV samples, whereas UAV-specific degradation simulation operations-motion blur simulation, sensor noise injection, JPEG compression emulation, and illumination normalization via CLAHE-are applied only to training samples as robustness-oriented augmentation.

An overview of the complete preprocessing workflow is illustrated in Figure 4, which summarizes the sequential transformations applied to each UAV frame. These stages include aspect-ratio-preserving resizing, pixel normalization, motion blur simulation, sensor noise modeling, JPEG compression emulation, and illumination normalization via CLAHE. Collectively, these operations work together to make sure that every input image is turned into a standardized and degradation-aware representation that preserves important forensic cues while making the system more robust against real-world UAV imaging problems. However, as clarified above, only resizing and normalization are universally applied to all dataset splits, whereas the degradation simulation operations are reserved for the training stage. The following subsections provide a detailed description of each preprocessing component, including mathematical formulas, theoretical justification, and algorithmic implementation, and how they are useful for detecting deepfakes in UAV environments.

#### 3.4.1. Image Resizing and Aspect Ratio Preservation

The camera angle, flight height, and field of view settings change, which gives VisDrone’s UAV images a lot of different native resolutions and aspect ratios. If you resize these frames without preserving their geometric structure, you could change the shapes of objects and how they relate to each other in space, and hide low-level forensic clues. Therefore, resizing must be carried out very carefully to keep the shapes the same. This means that the edges of objects, fine texture, and artifacts from manipulation stay the same. Because geometric consistency is required for fair comparison across dataset splits, this resizing stage is applied uniformly to the training, validation, and test sets as part of the universal preprocessing pipeline.

Bicubic interpolation was used to solve these problems. This method uses third-order polynomials for smooth resampling, which gives better results than simpler bilinear or nearest-neighbor methods. Forensic analysis requires a method that keeps the gradient continuous and does not cause aliasing because deepfake manipulation traces often show up along small gradient changes, micro-textures, and edge boundaries.

(1) $$I_{\mathrm{resized}}\left(x,y\right)=\sum_{i=-1}^{2}\sum_{j=-1}^{2}w\left(i,j\right)\;\cdot \;I\left(\frac{x}{s}-i,\frac{y}{s}-j\right)$$

The expression in Equation (1) defines a bicubic interpolation function used to compute the value of a pixel at coordinates (x,y) in the resized image [24]. The original image *I* is sampled at fractional coordinates (x/s,y/s), where *s* is the scaling factor chosen to preserve the aspect ratio. The weight coefficients w(i,j) represent cubic convolution constants that determine how surrounding pixel intensities will influence interpolation. This formulation ensures smoothness and avoids abrupt intensity transitions that might otherwise obscure or mimic forensic cues introduced by manipulation operations such as object insertion or diffusion-based inpainting. Preserving the authenticity of spatial structures while resizing is very important because deepfake manipulations do not always perfectly copy object geometry or keep micro-textures intact. The resizing mechanism expressed in Equation (1) allows subsequent forensic layers to amplify manipulation inconsistencies without dampening them.

An aspect-ratio-preserving resizing algorithm was used to standardize heterogeneous UAV frames into a fixed input tensor $I_{\mathrm{resized}}\in R^{256\times 256\times C}$ while explicitly preventing geometric distortion and preprocessing-induced forensic bias. Given a raw image $I\in R^{H\times W\times C}$, the procedure first extracts the original geometry via Shape(I) and computes a uniform scale factor s=min(256/H,256/W) so that both axes are resized proportionally, preserving object shapes, boundary geometry, and spatial relationships under UAV viewpoint variability. The scaled dimensions are then deterministically computed as $H^{\prime }=\max\left(1,\left\lfloor H\;\cdot \;s\right\rfloor \right)$ and $W^{\prime }=\max\left(1,\left\lfloor W\;\cdot \;s\right\rfloor \right)$ to avoid degenerate sizes, after which bicubic interpolation BicubicResize$\left(I,H^{\prime },W^{\prime }\right)$ produces $I_{\mathrm{scaled}}$, maintaining smooth gradient continuity and avoiding aliasing that could mask or mimic manipulation artifacts. To enforce a fixed 256×256 input regardless of the original resolution, the algorithm allocates a zero-initialized output canvas $I_{\mathrm{out}}$ = Zeros(256, 256, *C*) and computes centering offsets $x=\lfloor \left(256-H^{\prime }\right)/2\rfloor$ and $y=\lfloor \left(256-W^{\prime }\right)/2\rfloor$. If $H^{\prime }\leq 256$ and $W^{\prime }\leq 256$, the algorithm performs a centered *padding* case by copying pixels from $I_{\mathrm{scaled}}$ into the canvas using nested loops over indices (i,j), i.e., $I_{\mathrm{out}}\left[x+i,\;y+j,\;:\right]\leftarrow I_{\mathrm{scaled}}\left[i,j,:\right]$, ensuring consistent spatial alignment without stretching. Otherwise, it executes a centered *cropping* case by computing crop origins $x_{0}=\left\lfloor \left(H^{\prime }-256\right)/2\right\rfloor$ and $y_{0}=\left\lfloor \left(W^{\prime }-256\right)/2\right\rfloor$ and filling $I_{\mathrm{out}}\left[i,j,:\right]\leftarrow I_{\mathrm{scaled}}\left[x_{0}+i,\;y_{0}+j,:\right]$ for 0≤i,j≤255, thereby removing peripheral regions symmetrically while retaining the central context. After spatial standardization, intensity validity is enforced by clipping to a sensor-consistent range $I_{\mathrm{out}}\leftarrow$ ClipRange(*I*_out_, 0, 255) to eliminate interpolation-induced overflow/underflow, and the final normalization step $I_{\mathrm{resized}}\leftarrow$ Normalize(*I*_out_) maps the image into the training domain (e.g., [0,1] scaling or channel-wise standardization), improving numerical stability and convergence during optimization. Collectively, the pipeline components-Shape, scale computation, bicubic resampling, canvas allocation, conditional padding/cropping with explicit index-based assignment, clipping, and normalization-provide deterministic dimensionality, geometric fidelity, and photometric consistency, which are essential for robust UAV deepfake forensics, where manipulation traces must be learned independently of resolution and aspect ratio variability. As shown in Algorithm 1.
**Algorithm 1.** Aspect-Ratio-Preserving Resizing for UAV Image Standardization**Require:** Raw UAV image *I* **Ensure:** Standardized image $I_{\mathrm{resized}}\in R^{256\times 256\times C}$
1: **function** AspectResize(*I*, T=256**1. Extract Original Geometry and Compute Scaling**2:    (H,W,C)← Shape(I)3:    Compute uniform scale factor s←min(T/H,T/W) to prevent geometric distortion**2. Determine Scaled Dimensions**4:    $H^{\prime }\leftarrow \max\left(1,\left\lfloor H\;\cdot \;s\right\rfloor \right)$5:    $W^{\prime }\leftarrow \max\left(1,\left\lfloor W\;\cdot \;s\right\rfloor \right)$6:    Resize image using bicubic interpolation:7:    $I_{\mathrm{scaled}}\leftarrow$ BicubicResize$\left(I,\;H^{\prime },\;W^{\prime }\right)$**3. Allocate Fixed-Size Output Canvas**8:    Initialize zero array $I_{\mathrm{out}}\leftarrow$ Zeros(T,T,C)9:    Compute centering offsets $x\leftarrow \lfloor \left(T-H^{\prime }\right)/2\rfloor$, $y\leftarrow \lfloor \left(T-W^{\prime }\right)/2\rfloor$10:    **if** $H^{\prime }\leq T$ **and** $W^{\prime }\leq T$ **then***Insert scaled image into centered region (padding case)*11:        **for** i←0 **to** $H^{\prime }-1$ **do**12:           **for** j←0 **to** $W^{\prime }-1$ **do**13:               $I_{\mathrm{out}}\left[x+i,\;y+j,\;:\right]\leftarrow I_{\mathrm{scaled}}\left[i,j,:\right]$14:    **else***Extract central region to enforce fixed spatial dimensions (cropping case)*15:        $x_{0}\leftarrow \left\lfloor \left(H^{\prime }-T\right)/2\right\rfloor$16:        $y_{0}\leftarrow \left\lfloor \left(W^{\prime }-T\right)/2\right\rfloor$17:        **for** i←0 **to** T−1 **do**18:           **for** j←0 **to** T−1 **do**19:               $I_{\mathrm{out}}\left[i,j,:\right]\leftarrow I_{\mathrm{scaled}}\left[x_{0}+i,\;y_{0}+j,:\right]$**4. Enforce Intensity Validity and Normalize**20:    Clip pixel intensities to valid dynamic range:21:    $I_{\mathrm{out}}\leftarrow$ ClipRange$\left(I_{\mathrm{out}},\;0,\;255\right)$22:    Normalize pixel distribution for stable model training:23:    $I_{\mathrm{resized}}\leftarrow$ Normalize$\left(I_{\mathrm{out}}\right)$24:    **return** $I_{\mathrm{resized}}$25:$I_{\mathrm{resized}}\leftarrow$ AspectResize(I,256)26:**return** $I_{\mathrm{resized}}$


#### 3.4.2. Pixel Normalization and Standardization

After geometric normalization, it is necessary to convert the pixel intensity values into a common statistical domain. UAV images frequently display inconsistent exposure levels, resulting from variable illumination, shadowing, or sensor calibration drift. Failure to normalize such variation may cause the model to adopt spurious intensity correlations unrelated to forensic cues [25].

(2) $$I_{\mathrm{norm}}\left(x,y,c\right)=\frac{I_{\mathrm{resized}}\left(x,y,c\right)-\mu_{c}}{\sigma_{c}}$$

Equation (2) ensures that each color channel (R, G, B) has zero mean and unit variance based on the training set statistics $\mu_{c}$ and $\sigma_{c}$. Such standardization suppresses dataset-specific illumination biases and enhances the model’s sensitivity to subtle anomalies, including unnatural shading, color bleeding, and boundary inconsistencies, introduced during manipulation. Deepfake-generated content often fails to respect natural color distributions and cross-channel correlation, making normalization critical for highlighting these deviations.

#### 3.4.3. UAV Motion Blur Simulation

UAV platforms naturally create complicated motion patterns because they change direction quickly, vibrate from propellers, and suddenly speed up in translation. These dynamic movements often cause motion blur in photos, which shows up as directional smearing, edge streaking, and long gradients. Because VisDrone images are taken from different angles in the air, motion artifacts show up in different places in the dataset, making it hard to analyze the images visually because the blur patterns are not the same. Adding synthetic blur to preprocessing is necessary to make sure that the deepfake detection pipeline stays strong in real-world flight conditions. This synthetic blur must be carefully designed so that it does not destroy important forensic clues that could be used to find the manipulation signature. A realistic motion blur model must account for the directionality, blur length, and intensity distribution produced by UAV movement. In forensic contexts, motion blur interacts with digital manipulations in complex ways: it may mask edge inconsistencies introduced by tampering, distort texture continuity, or amplify differences between authentic and generated content. Therefore, accurately simulating blur is not a trivial augmentation step; rather, it is a sophisticated process that replicates UAV dynamics to expose the forensic model to authentic operational conditions. This reinforces the model’s ability to detect deepfakes in degraded environments where many existing detectors may fail [26].

(3) $$K_{\theta }\left(i,j\right)=\left\{\begin{array}{cc} \frac{1}{L}, & (i,j)\;\mathrm{lies}\;\mathrm{on}\;a\;\mathrm{linear}\;\mathrm{trajectory}\;\mathrm{of}\;\mathrm{length}\;L\;\mathrm{at}\;\mathrm{angle}\;\theta ,\\ 0, & \mathrm{otherwise}. \end{array}\right.$$

Equation (3) defines a linear motion blur kernel oriented at an angle θ and extending across *L* pixels. Conceptually, each kernel describes how a single point in an image spreads its intensity along a straight path, replicating the effect of camera movement during exposure. A constant 1/L ensures that the kernel integrates to one, preserving image brightness despite the redistribution of pixel intensities. By restricting non-zero values to positions lying exactly along a trajectory, the kernel model idealizes motion along a fixed direction, which corresponds closely to UAV forward motion or lateral drift during stable flight. These formulations are widely used in aerospace imaging simulations because they capture the directional consistency of the blur arising from deterministic movement patterns. In UAV forensic analysis, Equation (3) is highly relevant because synthetic deepfake regions may contain interpolation artifacts, misaligned edges, or incorrect textures that respond differently to motion blur. By applying a mathematically principled kernel $K_{\theta }$, the preprocessing pipeline ensures that blur affects both real and manipulated regions in a physically consistent manner, preventing the forensic model from identifying deepfakes based solely on blur inconsistencies. Instead, the model is encouraged to detect deeper structural or semantic anomalies that persist even after realistic motion degradation.

The UAV motion blur simulation algorithm models realistic aerial camera smear by generating a directionally controlled linear convolution kernel and applying adaptive quality constraints to preserve forensic integrity. Given a normalized input image $I_{\mathrm{norm}}\in R^{H\times W\times C}$, the procedure first samples the stochastic blur length L∈[5,20] and orientation $\theta \in [0^{\circ },180^{\circ })$ to emulate arbitrary UAV motion trajectories. A square kernel $K\in R^{L\times L}$ is initialized and populated along a discrete linear trajectory T derived from (L,θ), assigning equal weights 1/L along the motion direction before enforcing energy conservation via K←K/∑K. A padded image $I_{\mathrm{pad}}$ undergoes symmetric boundary extension to avoid edge truncation artifacts, after which spatial convolution produces an intermediate blurred tensor $I_{\mathrm{temp}}$, followed by intensity clipping to maintain numerical validity in [0,1]. To prevent excessive degradation of discriminative features critical for deepfake detection, an adaptive gradient preservation metric G is computed; if G exceeds the predefined tolerance $\tau_{\mathrm{grad}}$, the kernel length has to be reduced and the convolution is recomputed, thereby constraining structural information loss. An optional sharpening stage further compensates for over-smoothing when required. A second validation stage computes an edge-quality confidence score E, ensuring that high-frequency boundary structures are not excessively attenuated; if E falls below the tolerance $\tau_{\mathrm{edge}}$, the blur kernel is adaptively shortened and reapplied. Histogram normalization rebalances intensity distributions to maintain photometric consistency across the augmented samples. These components—stochastic parameter sampling, deterministic kernel construction, symmetric padding, controlled convolution, gradient-aware adaptation, edge validation, and histogram normalization—simulate realistic UAV motion blur while preserving the forensic cues necessary for robust deepfake discrimination. As shown in Algorithm 2.
**Algorithm 2.** UAV Motion Blur Simulation with Adaptive Quality Control**Require:** Normalized image $I_{\mathrm{norm}}\in R^{H\times W\times C}$ **Ensure:** Motion-blurred image $I_{\mathrm{blur}}\in R^{H\times W\times C}$
1: **function** ApplyMotionBlur($I_{\mathrm{norm}}$)**1. Initialize Blur Parameters**2:    (H,W,C)← Shape$\left(I_{\mathrm{norm}}\right)$3:    Sample kernel length L∼U{5,20}4:    Sample blur orientation $\theta ∼U(0^{\circ },180^{\circ })$**2. Construct Linear Motion Kernel**5:    Initialize kernel matrix K← Zeros(*L*, *L*)6:    Compute discrete linear trajectory T← LinearTrajectory(*L*, *θ*)7:    **for** each coordinate (i,j)∈T **do**8:        K[i,j]←1/L9:    Normalize kernel: $K\leftarrow K/\sum_{i,j}K\left[i,j\right]$**3. Apply Convolution with Symmetric Boundary Handling**10:    $I_{\mathrm{pad}}\leftarrow \leftarrow$ PadSymmetric$\left(I_{\mathrm{norm}}\right)$11:    $I_{\mathrm{temp}}\leftarrow$ Convolve$\left(I_{\mathrm{pad}},K\right)$12:    $I_{\mathrm{temp}}\leftarrow$ ClipRange$\left(I_{\mathrm{temp}},0,1\right)$**4. Adaptive Gradient Preservation Check**13:    G← GradientMetric$\left(I_{\mathrm{temp}}\right)$14:    **if** $G>\tau_{\mathrm{grad}}$ **then**15:        L←max(3,L−2)16:        Recompute *K* using updated *L*17:        $I_{\mathrm{temp}}\leftarrow$ Convolve$\left(I_{\mathrm{pad}},K\right)$**5. Optional Edge Restoration**18:    **if** RequiresSharpening$\left(I_{\mathrm{temp}}\right)$ **then**19:        $I_{\mathrm{temp}}\leftarrow$ Sharpen$\left(I_{\mathrm{temp}}\right)$**6. Final Edge Quality Validation**20:    $I_{\mathrm{blur}}\leftarrow$ MergeChannels$\left(I_{\mathrm{temp}}\right)$21:    E← EdgeQuality$\left(I_{\mathrm{blur}}\right)$22:    **if** $E<\tau_{\mathrm{edge}}$ **then**23:        L←max(3,L−3)24:        Recompute *K* and update $I_{\mathrm{blur}}$**7. Histogram Normalization**25:    $I_{\mathrm{blur}}\leftarrow$ HistNormalize$\left(I_{\mathrm{blur}}\right)$26:    **return** $I_{\mathrm{blur}}$27:$I_{\mathrm{blur}}\leftarrow$ ApplyMotionBlur$\left(I_{\mathrm{norm}}\right)$28:**return** $I_{\mathrm{blur}}$


#### 3.4.4. Sensor Noise Injection

UAV cameras frequently operate under challenging environmental conditions, including fluctuating temperatures, atmospheric scattering, and electronic interference induced by flight hardware. These conditions introduce different forms of image noise, such as Gaussian sensor noise, salt-and-pepper artifacts, and periodic banding. Deepfake regions, which are typically generated through smooth interpolation or GAN-based synthesis, often display unnatural noise patterns that differ from sensor-originated noise. Therefore, injecting noise into both real and synthetic images is crucial to prevent the forensic model from learning trivial differences in noise distribution. A realistic noise simulation helps expose the model to degradation patterns characteristic of UAV cameras, including photon shot noise, sensor heating effects, exposure inconsistencies, and intensity quantization. Without this simulation, the model would overfit to noise-free manipulations that rarely occur in real UAV operations. By incorporating stochastic noise components, the preprocessing pipeline ensures that the deepfake detector becomes invariant to superficial variations while remaining sensitive to meaningful manipulation artifacts.

(4) $$I_{\mathrm{gauss}}\left(x,y\right)=I_{\mathrm{blur}}\left(x,y\right)+N\left(0,\sigma_{n}^{2}\right)$$

Equation (4) describes the process of adding Gaussian noise to a blurred image. The term $N(0,\sigma_{n}^{2})$ represents a zero-mean Gaussian distribution with variance $\sigma_{n}^{2}$, modeling stochastic sensor fluctuations. Gaussian noise is commonly used to approximate electronic disturbances in image sensors, including thermal noise, readout noise, and quantization errors. The addition of this noise to a blurred image ensures that the resulting intensity distribution is consistent with what would be captured by UAV sensors during dynamic flight conditions. The equation reflects a foundational principle that noise must be additive and spatially uncorrelated, mimicking the random excitation of sensor wells. Gaussian noise plays a particularly important role in deepfake forensics because many generative manipulation methods fail to replicate realistic fine-grained noise structures. Authentic UAV images contain non-uniform noise patterns dependent on luminance, exposure time, and sensor sensitivity. In contrast, synthetic deepfake patches often display unnaturally smooth gradients or repetitive textures. By adding Gaussian noise using Equation (4), the preprocessing pipeline ensures that all regions—authentic or manipulated—exhibit a realistic noise floor, forcing the forensic model to rely on higher-level semantic and structural inconsistencies.

The UAV sensor noise modeling algorithm simulates realistic acquisition degradations by combining additive Gaussian perturbations, impulsive corruption, and adaptive variance control to approximate stochastic drone imaging behavior. Given a motion-blurred tensor $I_{\mathrm{blur}}\in R^{H\times W\times C}$, the procedure first samples the Gaussian noise level $\sigma_{n}∼U\left(0.01,0.05\right)$ and generates an additive noise tensor $G\in R^{H\times W\times C}$; these are applied element-wise and clipped to the valid intensity range [0,1] to maintain numerical stability. To emulate intermittent sensor dropouts, transmission corruption, and bit-flip artifacts common in UAV telemetry systems, a salt-and-pepper impulse process is applied per pixel and channel using the stochastic probability α∼U(0.001,0.01), injecting extreme intensity values in a controlled manner. A median filtering stage follows to prevent unrealistic pixel spikes while preserving structural boundaries. An algorithm then estimates the global residual variance V of a noisy frame; if V falls below the predefined tolerance τ, an adaptive reinforcement step has to increase $\sigma_{n}$ by Δσ and reintroduce Gaussian noise to ensure the frame matches the realistic stochastic variability observed in operational UAV imagery. A final postprocessing block applies Gaussian smoothing to simulate sensor diffusion effects, histogram alignment to prevent tonal drift across frames, and explicit edge-preservation filtering (Canny/Sobel-based) to ensure that high-frequency boundary cues—critical for deepfake artifact localization—are not excessively attenuated. These integrated components—Gaussian noise synthesis, impulse injection via nested loops, variance estimation, adaptive reinforcement, smoothing, histogram correction, and edge preservation—produce a photometrically and structurally consistent sensor noise profile that closely approximates real UAV imaging conditions while maintaining forensic discriminability. Algorithm 3 presents a details about UAV sensor noise modeling with adaptive variance control.
**Algorithm 3.** UAV Sensor Noise Modeling with Adaptive Variance Control**Require:** Motion-blurred image $I_{\mathrm{blur}}\in R^{H\times W\times C}$ **Ensure:** Sensor-realistic noisy image $I_{\mathrm{noise}}\in R^{H\times W\times C}$
1: **function** ApplySensorNoise($I_{\mathrm{blur}}$)**1. Initialize Dimensions and Gaussian Noise Parameters**2:    (H,W,C)← Shape$\left(I_{\mathrm{blur}}\right)$3:    Sample Gaussian variance $\sigma_{n}∼U\left(0.01,0.05\right)$4:    Generate additive noise tensor G← GaussianNoise$\left(H,W,C,\sigma_{n}\right)$5:    I← ClipRange$\left(I_{\mathrm{blur}}+G,\;0,\;1\right)$**2. Apply Impulsive Salt-and-Pepper Noise**6:    Sample impulse probability α∼U(0.001,0.01)7:    **for** x←1 **to** *H* **do**8:        **for** y←1 **to** *W* **do**9:           **for** c←1 **to** *C* **do**10:               r← Rand(0,1) 11:               **if** r<α/2 **then**12:                   I[x,y,c]←013:               **else if** r<α **then**14:                   I[x,y,c]←1**3. Median-Based Stabilization and Variance Estimation**15:    I← MedianFilter(I)16:    V← EstimateVariance(I)**4. Adaptive Noise Reinforcement**17:    **if** V<τ **then**18:        $\sigma_{n}\leftarrow \sigma_{n}+\Delta \sigma$19:        G← GaussianNoise$\left(H,W,C,\sigma_{n}\right)$20:        I← ClipRange(I+G,0,1)**5. Sensor-Realistic Postprocessing**21:    I← Smooth(I,GaussianKernel)22:    I← HistogramAlign(I)23:    I← PreserveEdges(I,Canny/Sobel)24:    **return** *I*25:$I_{\mathrm{noise}}\leftarrow$ ApplySensorNoise$\left(I_{\mathrm{blur}}\right)$26:**return** $I_{\mathrm{noise}}$


#### 3.4.5. JPEG Compression Simulation

Due to limited bandwidth, latency, and onboard resources, UAV systems often compress aerial frames before storing or sending them wirelessly. JPEG is popular because it uses an efficient block-based discrete cosine transform (DCT) compression technique. This compression creates artifacts that are unique to it, like blockiness, quantization errors, and a ringing effect. These artifacts frequently interact with manipulation traces; for instance, GAN-generated content may exhibit an incorrect DCT coefficient distribution, leading to discernible inconsistencies [27]. Simulating compression ensures that both real and manipulated frames undergo identical degradation, enabling the forensic model to remain robust under realistic operational conditions.

In forensic analysis, compression serves a dual function. On one hand, too much compression can hide deepfake artifacts by making texture irregularities less noticeable. Conversely, a lack of anticipated compression artifacts may itself signify manipulation. Adding controlled JPEG degradation ensures that DeepLayer-ID learns to find invariant forensic cues that stay the same no matter how much compression is carried out instead of relying on differences that happen because of compression.

(5) $$I_{\mathrm{jpeg}}=\mathrm{JPEG}\left(I_{\mathrm{noise}},q\right)$$

The operator in Equation (5) applies JPEG compression to a noise-enhanced image using the quality factor *q*. The JPEG function encapsulates several stages: conversion to YCbCr color space, segmentation into 8×8 blocks, application of 2D DCT, quantization using a *q*-dependent matrix, and entropy coding. The quantization stage introduces the most significant distortions because it reduces high-frequency information that is crucial for preserving fine textures and manipulation traces. The choice of the quality factor *q* determines how aggressively this high-frequency content is suppressed. From a forensic perspective, JPEG compression introduces block boundaries and coefficient truncation patterns that differ from those produced by generative synthesis. Deepfake content often lacks realistic DCT responses because GANs and diffusion models do not accurately model block-wise quantization. As a result, compression helps highlight inconsistencies between authentic and manipulated data. By applying a consistent compression operator, as defined in Equation (5), the preprocessing pipeline prevents the model from exploiting trivial JPEG-related differences, encouraging it to learn meaningful manipulation-sensitive features. Algorithm 4 presents a details about JPEG compression simulation with adaptive artifact control.
**Algorithm 4.** JPEG Compression Simulation with Adaptive Artifact Control**Require:** Noise-enhanced image $I_{\mathrm{noise}}\in R^{H\times W\times 3}$ **Ensure:** JPEG-compressed image $I_{\mathrm{jpeg}}\in R^{H\times W\times 3}$
1: **function** ApplyJPEGCompression($I_{\mathrm{noise}}$)**1. Color Space Transformation and Quality Initialization**2:    $I_{ycbcr}\leftarrow$ RGB2YCbCr$\left(I_{\mathrm{noise}}\right)$3:    Sample quality factor q∼U(40,90)4:    B← SplitIntoBlocks$\left(I_{ycbcr},\;8,\;8\right)$**2. Block-wise JPEG Encoding–Decoding Simulation**5:    **for** each block b∈B **do**6:        D←DCT2D(*b*)7:        Q← LoadQuantTable(q)8:        $C_{q}\leftarrow \mathrm{round}\left(D/Q\right)$9:        z← ZigzagScan$\left(C_{q}\right)$10:        r← RunLengthEncode(z)11:        h← HuffmanEncode(r)12:        $r^{\prime }\leftarrow$ HuffmanDecode(h)13:        $C_{q}\leftarrow$ RunLengthDecode$\left(r^{\prime }\right)$14:        $C\leftarrow C_{q}\;\cdot \;Q$15:        b←IDCT2D(*C*)**3. Image Reconstruction and Range Enforcement**16:    $I_{rec}\leftarrow$ Reconstruct(B)17:    $I_{rgb}\leftarrow$YCbCr2RGB(*I_rec_*)18:    $I_{rgb}\leftarrow$ ClipRange$\left(I_{rgb},\;0,\;1\right)$**4. Adaptive Compression Artifact Control**19:    M← ComputeBlockiness$\left(I_{rgb}\right)$20:    **if** M<τ **then**21:        q←max(10,q−Δq)22:        Recompute compression pipeline using updated *q*23:    **return** $I_{rgb}$24:$I_{\mathrm{jpeg}}\leftarrow$ ApplyJPEGCompression$\left(I_{\mathrm{noise}}\right)$25:**return** $I_{\mathrm{jpeg}}$


The JPEG compression simulation algorithm emulates the full block-based coding pipeline to reproduce realistic quantization artifacts encountered in UAV image transmission and storage systems. Given the noise-enhanced tensor $I_{\mathrm{noise}}\in R^{H\times W\times 3}$, the procedure first transforms an image into its luminance–chrominance domain via RGB2YCbCr, separating intensity from color components to mirror the JPEG standard. The stochastic quality factor q∼U(40,90) controls compression strength, after which the image is partitioned into non-overlapping 8×8 blocks B. For each block, a two-dimensional discrete cosine transform (DCT) produces a frequency coefficient *D*, which is quantized using the quality-dependent matrix *Q* through $C_{q}=\mathrm{round}\left(D/Q\right)$, thereby discarding high-frequency precision in a controlled manner. The coefficient is reordered via zigzag scanning to group low-frequency components first, then entropy-coded using run-length encoding and Huffman coding to simulate realistic compression bitstreams. A decoding process reconstructs quantized coefficients through inverse entropy operations before dequantization ($C=C_{q}\;\cdot \;Q$) and inverse DCT restore the spatial-domain blocks. After reconstructing the full image and converting back to RGB space, intensities are clipped to [0,1] to maintain numerical validity. To ensure that simulated compression produces measurable block-boundary artifacts, the blockiness metric M is computed; if M falls below the tolerance τ, the quality factor has to be reduced and the compression cycle is repeated to increase artifact strength. Collectively, the pipeline components—color space transformation, 8×8 block partitioning, DCT transformation, quantization, entropy coding/decoding, inverse reconstruction, and adaptive artifact control—faithfully reproduce the structural and frequency-domain distortions characteristic of operational JPEG compression, thereby providing realistic training conditions for UAV deepfake forensic analysis.

#### 3.4.6. Illumination Normalization via CLAHE

UAV imaging is very hard because of changing solar angles, shadows from buildings or moving objects, atmospheric haze, and reflective surfaces. Because of these things, the brightness is not the same across frames, making dark areas look too dark and bright areas look too bright. Deepfake manipulations frequently do not maintain consistent illumination, particularly in altered areas where shading gradients may be unnaturally smooth or misaligned with ambient lighting. So, it is necessary to normalize the lighting to improve local contrast and balance brightness while keeping forensic accuracy.

We used CLAHE contrast limited adaptive histogram equalization because it can improve local contrast in small grid areas without making noise too loud. Standard histogram equalization can change the overall brightness, but CLAHE uses adaptive processing to bring back details while keeping the lighting conditions realistic [28]. This technique enhances subtle texture patterns and micro-level inconsistencies, which are crucial in deepfake forensic analysis but may otherwise be obscured.

(6) $$I_{\mathrm{clahe}}=T_{\mathrm{CLAHE}}\left(I_{\mathrm{jpeg}}\right)$$

Equation (6) expresses a CLAHE transformation operator $T_{\mathrm{CLAHE}}$ that enhances local luminance by redistributing pixel intensities within small tile regions. During the process, each tile undergoes histogram equalization with a clip limit applied to prevent excessive amplification of noise spikes [29]. CLAHE operates in a LAB color space, modifying only the *L* luminance channel while preserving chrominance channels to avoid color distortion. This is particularly important for UAV forensic applications because chromatic consistency is a critical indicator of natural lighting. Applying CLAHE to JPEG-compressed images ensures that illumination inconsistencies are corrected while JPEG artifacts remain detectable [29]. Because deepfake manipulation methods often struggle to mimic realistic lighting behavior, a normalization step based on Equation (6) helps highlight contrast mismatches across manipulated patches. CLAHE improves the model’s ability to analyze boundary discontinuities, texture distribution, and structural shading cues that signify tampering.

### 3.5. DeepLayer-ID Model Architecture

The proposed DeepLayer-ID framework introduces a novel multi-branch forensic architecture that is meticulously engineered for detecting deepfake manipulations in UAV-based aerial surveillance environments. Distinct from conventional deepfake detectors that primarily target facial forgeries in ground-level imagery, DeepLayer-ID is tailored for aerial perspectives characterized by small-scale objects, motion-induced distortions, environmental noise, and heterogeneous backgrounds. These conditions significantly challenge traditional detectors by masking forgery traces beneath realistic UAV operational degradations.

**Figure 5:**
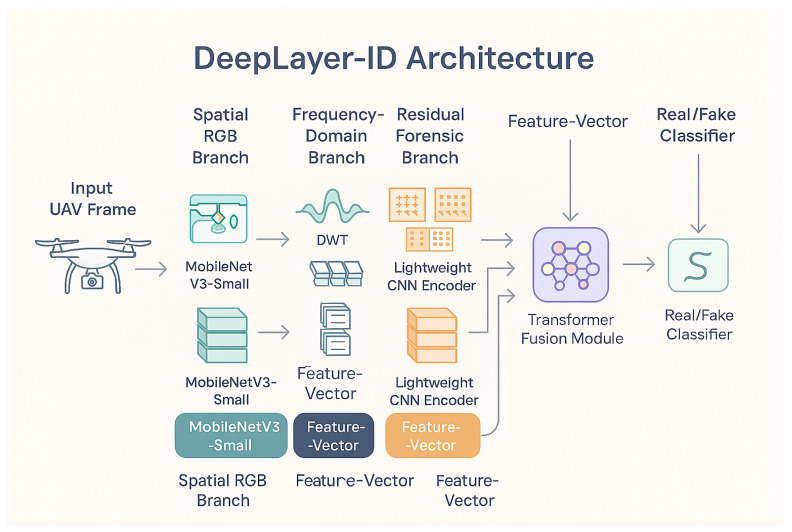
Overview of the proposed DeepLayer-ID architecture. The input UAV frame is processed through a spatial RGB branch, a DWT-based frequency-domain branch, and a residual forensic branch, whose feature vectors are fused via a transformer module for real/fake manipulation detection.

The design of the proposed DeepLayer-ID architecture is illustrated in Figure 5. The model processes each UAV frame through three complementary forensic branches to robustly capture manipulation cues embedded at different visual domains. A spatial RGB branch focuses on boundary textures and object geometry, while a frequency-domain branch highlights high-frequency inconsistencies introduced by GAN-based synthesis through DWT decomposition. At the same time, a residual forensic branch emphasizes noise-level and compression artifacts that manipulated regions often fail to replicate. The resulting feature representations are fused using a lightweight transformer module that enforces global semantic coherence across a scene before producing a final real/fake classification. This unified design enables DeepLayer-ID to remain highly effective under challenging UAV imaging conditions, such as under motion blur, variable illumination, and small-scale manipulated regions.

The transformer fusion module additionally provides implicit interpretability by exposing attention weights across feature domains, enabling analysis of how spatial, frequency, and residual cues contribute to the final decision.

**Figure 6:**
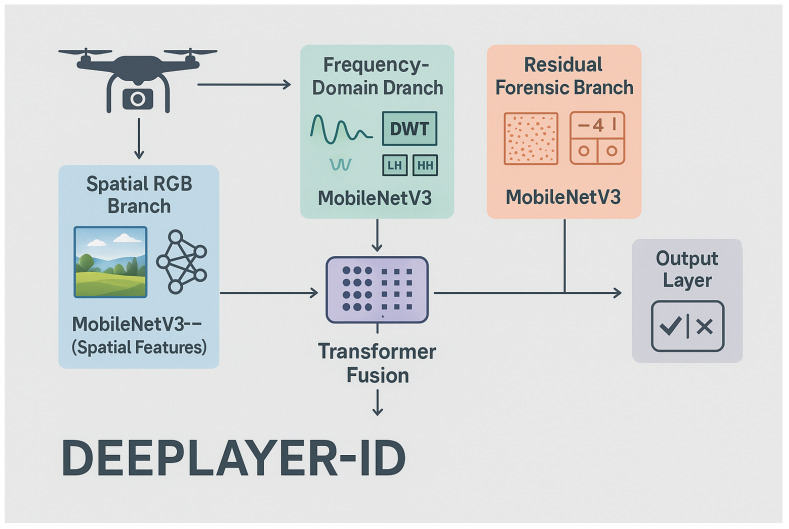
High-level DeepLayer-ID architecture. The input UAV frame is fed in parallel to three specialized branches.

DeepLayer-ID addresses these complexities through a hybrid design that synergistically combines lightweight convolutional backbones for spatial–local analysis with a transformer-based module for global contextual reasoning. A core motivation behind this architecture is rooted in the nature of UAV deepfake manipulations themselves: while local edits alter edge-level micro-textures and create high-frequency inconsistencies, sophisticated generative models attempt to preserve global structural coherence to evade detection. Therefore, a successful forensic detector must simultaneously capture localized anomaly cues and enforce global semantic consistency constraints, capabilities offered collectively by CNNs and transformers. As illustrated in Figure 6, the proposed DeepLayer-ID architecture adopts a multi-branch design in which an input UAV frame is simultaneously processed by three complementary forensic streams. The spatial RGB branch focuses on dense object textures and geometric boundaries, the frequency-domain branch isolate high-frequency wavelet components, and the residual forensic branch emphasize sensor- and compression-related noise patterns. Their latent representations are then aligned and fused by a multi-head transformer module that enforces scene-level semantic consistency before producing a binary real/fake decision.

- Spatial RGB Branch: A MobileNetV3-Small backbone extracts texture continuity, object-edge fidelity, and geometric consistency in authentic regions. This ensures sensitivity to the boundary-level tampering that commonly accompanies adversarial object insertion or removal.
- Frequency-Domain Branch: Two-dimensional Discrete Wavelet Transform (DWT) decomposes a frame into directional sub-bands (LH, HL, HH), explicitly isolating high-frequency forensic artifacts often left behind by diffusion-based synthesis and patch blending operations. This branch is capable of revealing subtle anomalies invisible in a spatial domain.
- Residual Forensic Branch: A hybrid SRM + Laplacian high-pass filter bank enhances noise residuals while suppressing semantic content. Since manipulated textures frequently fail to replicate sensor-specific noise patterns or compression response characteristics, this branch exposes discrepancies that generative models attempt to conceal.

**Figure 7:**
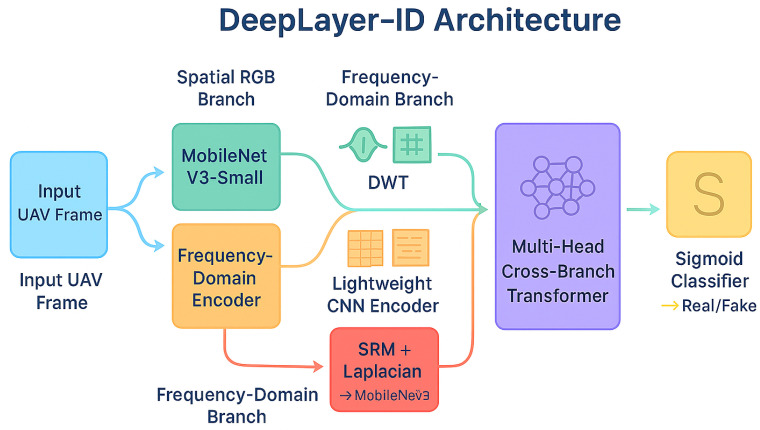
Three-dimensional data-flow representation of DeepLayer-ID. The preprocessed UAV frame is dispatched to a spatial RGB encoder, a DWT-based frequency encoder, and a residual noise encoder. Their feature vectors are concatenated and refined via a multi-head cross-branch transformer, whose output is fed to a sigmoid classifier that yields the final real/fake decision. This figure emphasizes this parallel feature extraction and subsequent transformer-based fusion.

Figure 7 provides a more detailed view of the internal data flow across the three branches and the transformer fusion unit. Starting from a preprocessed UAV frame, spatial, frequency, and residual cues are extracted by the three MobileNetV3-based encoders and projected into a common latent space. A transformer block then performs cross-branch attention, allowing the model to jointly reason about local manipulation traces and global scene context before passing a fused embedding to a sigmoid classifier. This layout highlights how DeepLayer-ID integrates complementary forensic evidence rather than relying on a single-domain representation.

To improve the interpretability of the transformer-based fusion mechanism, we further analyze the contribution of each feature domain through branch-wise attention visualization, as illustrated in Figure 7. The RGB branch primarily responds to spatial inconsistencies such as object boundaries, geometric distortions, and semantic anomalies. In contrast, the DWT-based frequency branch highlights high-frequency irregularities, including texture smoothing and compression artifacts, which are often introduced during generative manipulation. The residual forensic branch focuses on noise-level discrepancies and sensor-related inconsistencies, which are difficult for generative models to reproduce accurately. The fused attention map produced by the transformer demonstrates that the model adaptively weights these domains depending on the type of manipulation. For example, in cases of object insertion or removal, stronger attention is observed in the RGB and DWT branches, while identity-level or subtly blended manipulations trigger higher activation in the residual branch. This behavior indicates that the transformer does not rely on a single feature domain, but instead learns a context-aware fusion strategy that exploits complementary forensic cues across spatial, frequency, and noise representations. These observations confirm that the cross-domain attention mechanism is both meaningful and interpretable, as it aligns with known characteristics of deepfake generation artifacts in UAV imagery.

In contrast to single-stream detectors that risk overfitting to superficial visual differences or dataset-specific biases, DeepLayer-ID achieves a cross-domain anomaly consensus. Features extracted from the three independent streams are subsequently aligned and fused using a multi-head cross-branch transformer encoder [30]. This fusion mechanism enforces the following: (1) Long-range relational consistency across objects and background structures; (2) Global contextual verification of forged regions; (3) Simultaneous penalization of spatial-, frequency-, and noise-domain irregularities.

Integration of CNNs with transformer fusion allows DeepLayer-ID to reason holistically about scene integrity, a crucial advantage in UAV surveillance where tampered objects such as vehicles, pedestrians, and structural elements must remain consistent with their surroundings in terms of geometry, shadow orientation, illumination, and depth layer interaction.

The proposed DeepLayer-ID framework is purposefully engineered to overcome the unique forensic challenges present in UAV-based aerial surveillance imagery. Unlike conventional deepfake detection models that rely solely on global texture patterns, DeepLayer-ID explicitly captures fine-grained manipulation traces through a spatial RGB branch that focuses on dense, small-scale objects susceptible to occlusion and motion distortions. To detect subtle spectral inconsistencies introduced by GAN- and diffusion-based synthesis techniques, the frequency-domain DWT branch isolates high-frequency components that are often overlooked in the RGB domain. Furthermore, the residual forensic branch highlights abnormal sensor noise characteristics, illumination deviations, and compression footprints that frequently accompany synthetic insertions. Finally, the transformer-based fusion module verifies semantic coherence by modeling long-range dependencies within a scene, exposing context-level anomalies such as incorrect object scale, shadow alignment, and depth relationships. By jointly leveraging spatial, spectral, and residual domain cues, DeepLayer-ID demonstrates superior robustness and generalization against sophisticated manipulation attempts in realistic UAV surveillance environments.

Table 3 summarizes the split-aware input preparation protocol adopted in DeepLayer-ID. This table distinguishes between universal preprocessing operations, which are required to standardize all samples before feature extraction, and degradation-aware augmentation operations, which are introduced only during training to improve robustness under realistic UAV sensing conditions. In particular, resizing and normalization are applied uniformly across the training, validation, and test sets because they define the common spatial and statistical input space of the model. By contrast, motion blur, sensor noise, JPEG compression, and CLAHE-based illumination adjustment are treated as robustness-oriented training perturbations with explicit operational motivation: they emulate UAV motion dynamics, sensor instability, transmission artifacts, and luminance variability, respectively. Their exclusion from the validation and test sets is intentional, since held-out samples must remain free from artificial augmentation in order to provide a methodologically consistent estimate of true generalization performance.

#### 3.5.1. Mathematical Formulation of Branch Operations

This subsection presents a mathematical representation of the DeepLayer-ID feature extraction pipeline and explains how each component contributes to deepfake detection in UAV imagery. Let $I_{\mathrm{final}}$ denote a preprocessed UAV frame after standardization and UAV-specific degradation modeling. A first operation applies a MobileNetV3-Small convolutional encoder to learn spatially discriminative patterns such as human silhouettes, edge contours, and texture irregularities, producing spatial features $F_{\mathrm{RGB}}$ according to [31] as follows:

(7) $$F_{\mathrm{RGB}}=f_{\mathrm{MobileNetV}3}\left(I_{\mathrm{final}}\right),$$

where $f_{\mathrm{MobileNetV}3}$ denotes a lightweight convolutional backbone. To further expose the high-frequency inconsistencies introduced by generative models, a 2D discrete wavelet transform (DWT) decomposes an input into four orthogonal sub-bands, expressed as

(8) $$\left\{LL,LH,HL,HH\right\}=\mathrm{DWT}\left(I_{\mathrm{final}}\right),$$

where only high-frequency sub-bands LH, HL, and HH are retained for further encoding. These are concatenated and processed through a second MobileNetV3 encoder to yield $F_{\mathrm{DWT}}$, as follows:

(9) $$F_{\mathrm{DWT}}=f_{\mathrm{MobileNetV}3}\left(\mathrm{Concat}(LH,HL,HH)\right),$$

Noise-domain forensic evidence is isolated using a hybrid filter bank composed of a Spatial Rich Model (SRM) and Laplacian kernels [32]. The residual responses emphasize subtle anomalies in noise propagation, illumination continuity, and compression patterns as follows:

(10) $$R\left(x,y\right)=I_{\mathrm{final}}*\left\{K_{\mathrm{SRM}}^{\left(1:3\right)},K_{\mathrm{Laplace}}\right\},$$

and the resulting residual maps are encoded into deep residual embeddings $F_{\mathrm{Res}}$ by a same convolutional backbone, as follows:

(11) $$F_{\mathrm{Res}}=f_{\mathrm{MobileNetV}3}\left(R\right).$$

Three heterogeneous feature sets are then fused to support cross-modal reasoning through transformer-based multi-head self-attention. The initial fused representation is formed as

(12) $$Z_{0}=\mathrm{Concat}\left(F_{\mathrm{RGB}},F_{\mathrm{DWT}},F_{\mathrm{Res}}\right),$$

and refined by attention weighting as follows:

(13) $$\mathrm{MHSA}\left(Q,K,V\right)=\mathrm{Softmax}\left(\frac{QK^{T}}{\sqrt{d_{k}}}\right)V,$$

This ensures that long-range semantic relationships remain consistent throughout the scene. Binary manipulation decisions are obtained by applying Global Average Pooling (GAP) followed by a sigmoid-activated linear classifier, as follows:

(14) $$\hat{y}=\sigma \left(W_{\mathrm{cls}}\;\cdot \;\mathrm{GAP}\left(Z_{N}\right)+b_{\mathrm{cls}}\right),$$

where $Z_{N}$ denotes a fused representation after *N* transformer blocks, yielding a probabilistic prediction of whether the UAV frame is real or manipulated. Through this multi-domain formulation, DeepLayer-ID combines complementary evidence from spatial, frequency, and residual perspectives to deliver robust and context-aware deepfake detection in aerial surveillance environments.

#### 3.5.2. Training, Data Preparation, and Computational Strategy

**Figure 8:**
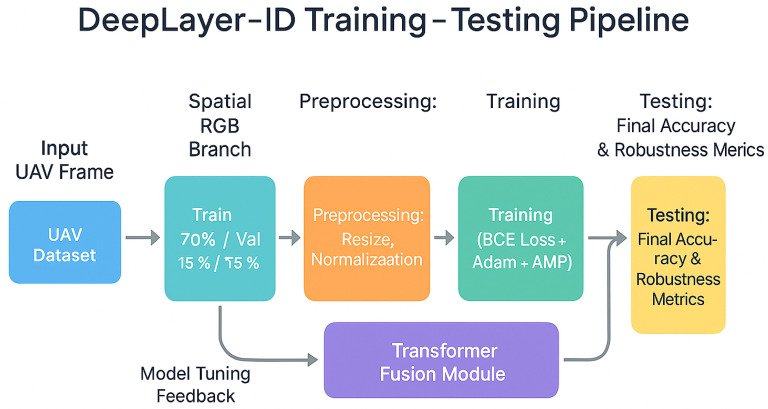
Training and testing pipeline for DeepLayer-ID.

This subsection details the training pipeline, dataset preparation, and computational footprint required to reproduce our DeepLayer-ID framework. A unified view of the end-to-end optimization is formalized through both mathematical formulation and procedural algorithms. Model implementation was conducted using PyTorch 2.1 with CUDA-enabled GPU acceleration. Computation was performed on an NVIDIA RTX 4090 GPU (24 GB VRAM), where Automatic Mixed Precision (AMP) training is enabled to lower memory load and accelerate matrix operations by up to 40% without compromising detection fidelity. The complete training and evaluation workflow of DeepLayer-ID is summarized in Figure 8. The balanced UAV dataset is first partitioned into training, validation, and test splits using a 70%/15%/15% protocol. All samples are then passed through a UAV-specific preprocessing pipeline, which standardizes resolution and simulates operational degradations. The processed training set drives optimization of the DeepLayer-ID parameters using BCE loss, Adam, and AMP, while the validation split is used for early stopping and learning-rate scheduling. After convergence, the held-out test split is used to report final accuracy and robustness metrics under realistic aerial conditions [33]. This dataset scale is also consistent with the lightweight design objective of DeepLayer-ID, which emphasizes efficient UAV deployment and robust learning from a balanced, domain-specific corpus rather than reliance on very large-scale training data. Figure 8 presents a details about the training and testing pipeline for DeepLayer-ID.

It should be noted that the current computational evaluation focuses on algorithmic efficiency metrics obtained on the experimental platform, whereas direct onboard power profiling under real UAV flight conditions is left for future deployment-oriented validation.

Binary manipulation detection was optimized using Binary Cross-Entropy (BCE) [34], as follows:

(15) $$L_{\mathrm{BCE}}=-\frac{1}{N}\sum_{i=1}^{N}\left[y_{i}\log\left({\hat{y}}_{i}\right)+\left(1-y_{i}\right)\log\left(1-{\hat{y}}_{i}\right)\right]$$

This loss penalizes misclassification of real and manipulated samples by enforcing probabilistic separation of prediction ${\hat{y}}_{i}$ from ground truth $y_{i}$ for *N* training samples.

Algorithm 5 outlines the training process for DeepLayer-ID, where real and manipulated UAV samples are used to compute prediction errors via BCE loss. An Adam optimizer updates model weights with gradient clipping to ensure stability, while AMP accelerates computations in an inconsistent way. A cosine annealing scheduler adjusts the learning rate for better convergence, and early stopping prevents overfitting by monitoring validation performance. The result becomes the optimized DeepLayer-ID model, ready for evaluation on unseen UAV data.
**Algorithm 5.** Training Loop for DeepLayer-ID Optimization**Require:** Training set $D_{train}$, validation set $D_{val}$, maximum epochs *T* **Ensure:** Optimized model parameters $\theta^{★}$
**Initialize**
1:(θ,η)←InitializeParameters()**Epoch-wise Optimization**2: **for** t=1 to *T* **do**3:    $\left(I,y\right)\leftarrow \mathrm{SampleMiniBatch}\left(D_{train}\right)$4:    $\hat{y}\leftarrow f_{\theta }\left(I\right)$                    ▹ forward pass5:    $L\leftarrow L_{\mathrm{BCE}}\left(y,\hat{y}\right)$              ▹ loss computation6:    $\nabla_{\theta }\leftarrow \nabla_{\theta }L$                  ▹ backpropagation7:    $\theta \leftarrow \mathrm{AdamUpdate}(\theta ,\nabla_{\theta },\eta )$   **Stability Enhancements**8:    θ←GradientClip(θ)9:    θ←AMPUpdate(θ)   **Validation and Scheduling**10:    $L_{val}\leftarrow \mathrm{Validate}\left(f_{\theta },D_{val}\right)$11:    η←CosineAnnealing(η,t,T)12:    **if** $\mathrm{EarlyStop}(L_{val})$ **then**13:        **break**14:$\theta^{★}\leftarrow \theta$15:**return** $\theta^{★}$


Table 4 summarizes the hyperparameters that were empirically found to maximize validation accuracy and generalization across UAV manipulation patterns. Our dataset comprises 1096 UAV images, including 548 authentic VisDrone2019-DET-val frames and 548 manipulated counterparts. Stratified sampling ensures a balanced protocol.

(16) Train:Val:Test=70%:15%:15%

Ensuring identical exposure of manipulation types and scene settings across splits, all frames underwent a UAV-specific degradation simulation to preserve operational realism, as follows:

- Bicubic resizing to 256×256;
- Mean-variance normalization;
- UAV motion blur emulation;
- Gaussian and impulse noise injection;
- JPEG compression with q∈[40,90];
- CLAHE illumination normalization.

Algorithm 6 outlines a split-aware input preparation pipeline for UAV imagery that ensures consistent spatial resolution and pixel normalization across all dataset subsets. Aspect-ratio-preserving resizing and RGB intensity normalization are applied to the training, validation, and test sets as universal preprocessing operations. In contrast, motion blur simulation, sensor noise injection, JPEG compression emulation, and CLAHE-based illumination normalization are conditionally applied only to the training set as degradation-aware augmentation steps are intended to improve robustness under realistic UAV acquisition conditions. This distinction ensures that DeepLayer-ID is trained to handle operational aerial degradations while preserving an unbiased and methodologically consistent evaluation protocol for the validation and test samples.
**Algorithm 6.** Split-Aware Preprocessing and Training-Only UAV Degradation Simulation**Require:** Raw UAV image $I_{\mathrm{raw}}$, dataset split S∈{train,val,test} **Ensure:** Processed sample $I_{\mathrm{final}}$    **Step 1: Aspect-Ratio-Preserving Resize**
1:$I\leftarrow \mathrm{resize}\_\mathrm{bicubic}(I_{\mathrm{raw}},\;\left(256256\right))$**Step 2: RGB Intensity Normalization**2:I←normalize(I)**Step 3: Training-Only UAV Degradation Simulation**3: **if** S=train **then**4:    I←simulate_motion_blur(I)5:    I←inject_sensor_noise(I)              ▹ Gaussian + salt-pepper6:    I←jpeg_compress(I)7:    I←apply_CLAHE(I)8:**end if****Step 4: Output Processed Sample**9:$I_{\mathrm{final}}\leftarrow I$10:**return** $I_{\mathrm{final}}$


Table 5 confirms the sample integrity and class-balance enforcement critical for unbiased training. The dataset consists of a balanced number of real and fake samples, which is essential to prevent model bias toward either class. Maintaining an equal ratio of 1:1 ensures that the model learns discriminative features effectively without favoring this category. Additionally, a consistent resolution of 256×256 pixels was standardized across the input data, simplifying preprocessing and reducing computational overhead. The absence of required annotations further streamlines the training process, focusing on binary classification rather than detailed labeling. A leakage check is important because it makes sure that there is no overlap between the training, validation, and test splits. This protects against data leakage, which could make model performance look better than it really is. All of these dataset features work together to create a strong base for training models that are reliable and can be used in many different situations. Deeplayer-id preserves real-time inference feasibility while expanding forensic sensitivity. Model complexity is reported per branch.

Table 6 importantly points out that the model keeps a small footprint that is good for embedded UAV processors. Even though it has more forensic features, the deepLayer-ID architecture needs to keep a good balance between model complexity and inference speed. Each branch is optimized to reduce the amount of computation needed, making sure that the total latency stays below 10 ms. This low latency is very important for real-time applications, like on-board UAV detection systems, where quick decision-making is becoming more and more important. Furthermore, a small number of parameters and flops makes it possible to run on hardware with limited resources without losing detection accuracy. This makes these deepLayer-IDs very useful for real-world forensic and surveillance tasks.

DeepLayer-ID is unique because it combines local spectral noise analysis with global semantic validation. This combination was not present in the baselines that were compared in Table 7. This table shows a comparison of capabilities against baseline architectures, with deepLayer-ID consistently performing well across all evaluated capabilities. For real-time 4k streaming, multi-GPU scaling may be needed. Under extreme compression, both real and fake content will degrade at the same rate, making it harder to separate them in the forensic investigation.

For fairness, all baseline models used in the comparative evaluation, including ResNet-50, XceptionNet, and Noiseprint CNN, were retrained using the same train/validation/test split and the same UAV-specific preprocessing pipeline applied to DeepLayer-ID. This preprocessing includes aspect-ratio-preserving resizing, motion blur simulation, sensor noise injection, JPEG compression emulation, and CLAHE-based illumination normalization. Therefore, the reported comparison reflects differences in model architecture and feature-learning capabilities rather than inconsistencies in data preparation or degradation exposure.

The transformer-based fusion module is implemented as a lightweight encoder designed to integrate multi-domain feature representations from the RGB, DWT, and residual branches. Specifically, the fused feature tensor is first projected into a shared embedding space of dimension *d*, after which it is processed by $N_{t}$ stacked transformer encoder blocks. Each block consists of multi-head self-attention with *H* attention heads, followed by a position-wise feed-forward network of dimension $d_{ff}$, residual connections, layer normalization, and dropout regularization with rate *p*. The input tokens correspond to the concatenated feature embeddings extracted from the three branches, allowing the attention mechanism to model cross-domain dependencies between spatial, frequency, and noise-level representations. This design enables the fusion module to selectively emphasize complementary forensic cues while suppressing redundant information. Despite its expressive capability, the module remains computationally efficient due to its shallow depth and reduced embedding dimensionality, contributing to only a small fraction of the total model complexity, as reflected in the parameter and FLOP analysis reported in Table 8.

The proposed DeepLayer-ID framework is intended for deployment on resource-constrained edge AI platforms typically used in UAV systems, such as embedded GPU or AI-accelerator modules. Although the reported 9.8 ms latency corresponds to the evaluation environment used in this study, the model was intentionally designed with a lightweight footprint (5.4 M parameters and 1.43 GFLOPs) to facilitate practical migration to onboard platforms. In such settings, the expected runtime may increase relative to the reported measurement; however, the compact architecture makes near-real-time and real-time inference feasible for UAV surveillance applications, particularly when optimized inference engines are employed.

### 3.6. Scope of Analysis and Temporal Modeling Boundaries

The proposed DeepLayer-ID framework is formulated as a frame-wise UAV forensic detection architecture in which each preprocessed aerial image of size 256×256×3 is analyzed independently through three complementary representational streams. The first stream is a spatial RGB branch based on MobileNetV3-Small that learns fine-grained texture continuity, object boundary fidelity, local geometric structure, and scene-level semantic patterns that may be disrupted by object insertion, object removal, facial identity manipulation, or synthetic region blending. The second stream is a DWT-based frequency branch, where the input frame is decomposed into the LL, LH, HL, and HH sub-bands, and the high-frequency components are encoded to expose spectral energy irregularities, synthesis-induced smoothing, high-frequency attenuation, and blending-related discontinuities that are often difficult to observe reliably in the RGB domain alone. The third stream is a residual forensic branch that applies SRM and Laplacian high-pass filtering to isolate sensor noise inconsistencies, compression response abnormalities, and local residual traces that generative manipulation pipelines often fail to reproduce faithfully under realistic UAV acquisition conditions. The resulting feature embeddings are projected into a shared latent space and fused by a lightweight transformer encoder configured with $N_{t}=2$ encoder blocks, H=4 attention heads, embedding dimension d=256, feed-forward dimension $d_{ff}=512$, and dropout rate p=0.1, after which a sigmoid classifier produces the final frame-level real/fake prediction. This design preserves strong forensic sensitivity across spatial, spectral, and residual domains while maintaining a computational efficiency suitable for resource-constrained UAV sensing platforms, with an overall complexity of 5.4 M parameters, 1.43 GFLOPs, and approximately 9.8 ms inference latency.

Within this formulation, the forensic analysis is centered on intra-frame evidence and on the consistency of manipulation-sensitive cues observable within an individual UAV image. Such cues include boundary discontinuities, texture inconsistency, sub-band energy distortion, residual noise mismatch, compression-related traces, illumination irregularity, and scene-level semantic incoherence arising from synthetic editing operations. This scope is consistent with the adopted data construction and training protocol, where the dataset consists of 1096 balanced UAV frames (548 authentic and 548 manipulated) partitioned using a stratified train/validation/test split of 70%/15%/15% and processed through a degradation-aware pipeline involving aspect-ratio-preserving resizing, pixel normalization, motion blur simulation, sensor noise injection, JPEG compression emulation, and CLAHE-based illumination normalization. At the same time, temporal consistency is defined here as a related but broader analytical dimension that involves relationships across consecutive frames, such as inter-frame flickering, temporal identity drift, motion continuity violations, trajectory inconsistency, object persistence anomalies, and cross-frame semantic instability. Since these effects emerge through sequential frame interactions rather than isolated frame content, they are outside the direct representational scope of the present architecture.

### 3.7. Evaluation Metrics

To ensure a comprehensive and unbiased assessment of the proposed deepLayer-ID framework, multiple performance evaluation metrics were adopted to quantify detection accuracy, robustness, and reliability under real-world UAV surveillance conditions. Because a task be formulated as a binary classification problem, distinguishing authentic UAV imagery from synthetically manipulated deepfake frames, this evaluation places particular emphasis on error types that may critically influence operational decision-making in security-sensitive environments. These evaluation metrics follow standard formulations widely used in machine learning and classification theory [35,36,37]. Let TP, TN, FP, and FN denote true positives, true negatives, false positives, and false negatives, respectively. The key metrics used in this study are defined below.

1. Accuracy

Accuracy reflects the overall proportion of correct predictions made by the model across real and manipulated cases [35]:

(17) $$\mathrm{Accuracy}=\frac{TP+TN}{TP+TN+FP+FN}.$$

2. Precision

Precision quantifies the reliability of positive manipulated predictions [35]:

(18) $$\mathrm{Precision}=\frac{TP}{TP+FP}.$$

3. Recall (Detection Sensitivity)

Recall measures the proportion of actual manipulated frames successfully detected [35]:

(19) $$\mathrm{Recall}=\frac{TP}{TP+FN}.$$

4. F1-Score

The f1-score balances precision and recall via the harmonic mean [35]:

(20) $$F1-\mathrm{Score}=2\;\cdot \;\frac{\mathrm{Precision}\;\cdot \;\mathrm{Recall}}{\mathrm{Precision}+\mathrm{Recall}}.$$

5. Receiver Operating Characteristic and AUC

The ROC curve illustrates the relationship between the true positive rate (TPR) and false positive rate (FPR) widely used in detection and verification tasks [37], as follows:

(21) $$\mathrm{TPR}=\frac{TP}{TP+FN},\;\mathrm{FPR}=\frac{FP}{FP+TN}.$$

The area under the curve (AUC) summarizes overall classifier separability [37]:

(22) $$\mathrm{AUC}=\int_{0}^{1}\mathrm{TPR}\left(\mathrm{FPR}\right)\;d\left(\mathrm{FPR}\right).$$

Supplemental numerical evaluation visual confusion matrices are employed to analyze class-wise performance under varying levels of scene complexity, motion blur, and deepfake manipulation types. This diagnostic representation helped identify potential biases and failure modes that would arise when synthetic regions were small, heavily occluded, or spectrally similar to their surrounding context.

## 4. Results and Discussion

This section presents a comprehensive empirical evaluation of deepLayer-ID on unseen UAV imagery, following the experimental protocol described in Section 3. We first quantify overall manipulation detection performance on a held-out test split using the accuracy, precision, recall, f1-score, and AUC metrics that were defined in the methodology. We then analyze robustness with respect to different manipulation styles and UAV-specific degradations, including motion blur, sensor noise, JPEG compression, and illumination variation. Next, we perform ablation studies to isolate the contribution of the spatial RGB branch, the DWT-based frequency branch, the residual forensic branch, and the transformer-based fusion module. We further benchmark deepLayer-ID against representative CNN- and forensics-oriented baselines and examine its computational complexity, runtime throughput, and memory footprint in the context of real-time UAV deployment. Finally, we provide an operational interpretation of these observed error modes and qualitative forensic behavior, highlighting scenarios in which the model would be most and least reliable.

Despite the strong performance achieved on the constructed dataset, it is important to acknowledge that the dataset size remains limited relative to large-scale real-world UAV deployments. In such scenarios, long-tail conditions, rare object categories, and previously unseen manipulation patterns may introduce additional challenges, potentially leading to false positives or false negatives. In particular, rare targets, extreme viewpoints, and highly subtle manipulations may not be fully represented in the current dataset, which can affect generalization under operational conditions. To mitigate this limitation, the proposed framework incorporates multiple complementary feature domains (spatial, frequency, and residual), as well as a degradation-aware preprocessing pipeline, both of which are designed to improve robustness beyond the training distribution. Nevertheless, future work will focus on extending the dataset with larger-scale UAV imagery, more diverse manipulation types, and long-tail scenario coverage to further enhance real-world reliability.

The current study focuses on RGB-based UAV imagery and does not explicitly evaluate deepfake detection for infrared (IR), low-light, or multispectral sensors. This is an important consideration because different sensing modalities exhibit distinct noise characteristics, radiometric responses, and spectral signatures, which can alter the appearance of both authentic and manipulated regions. Consequently, forgery cues learned from RGB data may not directly transfer to other sensor domains. Nevertheless, several components of the proposed DeepLayer-ID framework are modality-agnostic. In particular, the frequency-domain (DWT) and residual branches capture high-frequency inconsistencies and noise-level discrepancies that are not strictly tied to RGB color information, suggesting potential applicability across different sensor types. Furthermore, the degradation-aware preprocessing pipeline can be adapted to simulate modality-specific effects (e.g., thermal noise in IR or extreme noise in low-light conditions). Future work will extend the proposed framework to multimodal UAV datasets, including infrared, low-light, and multispectral imagery, and will investigate cross-sensor generalization through domain adaptation and multi-branch modality-aware training.

### 4.1. Overall Manipulation Detection Performance

DeepLayer-ID demonstrates a strong capability in distinguishing authentic UAV imagery from manipulated content on the held-out test split. As summarized in Table 9, the model attains an accuracy of 97.8%, precision of 98.1%, recall of 97.3%, and balanced f1-score of 97.7%. These metrics are computed according to the definitions and formulas introduced in Section 3 and jointly indicate that deepLayer-ID was effective in detecting forged frames while keeping false alarms at a low level. The corresponding AUC of 0.991 further confirms the near-perfect probabilistic separability between real and synthetic UAV data, reflecting the model’s ability to maintain a clear decision margin under diverse operational conditions.

**Figure 9:**
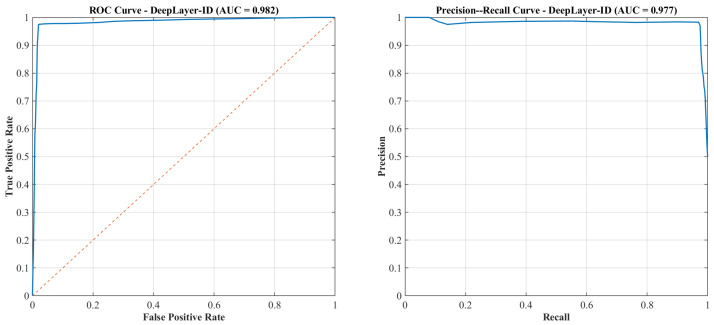
(**Left**) ROC curve of DeepLayer-ID on the test set, with AUC = 0.991. (**Right**) Precision–recall curve, illustrating threshold-dependent trade-offs between missed detections and false alarms.

The confusion matrix in Figure 9 provides further insight into the error distribution. False negatives predominantly arise in identity-based micro-manipulations where extremely small or partially occluded facial regions are altered in ways that challenge both the spatial and residual branches. Conversely, false positives were mainly observed under severe JPEG compression or pronounced motion blur, where block artifacts and texture smearing resembled the synthetic generative priors. Despite these challenging edge cases, a diagonal dominance of the confusion matrix indicates stable generalization and negligible drift between the training and testing distributions.

**Figure 10:**
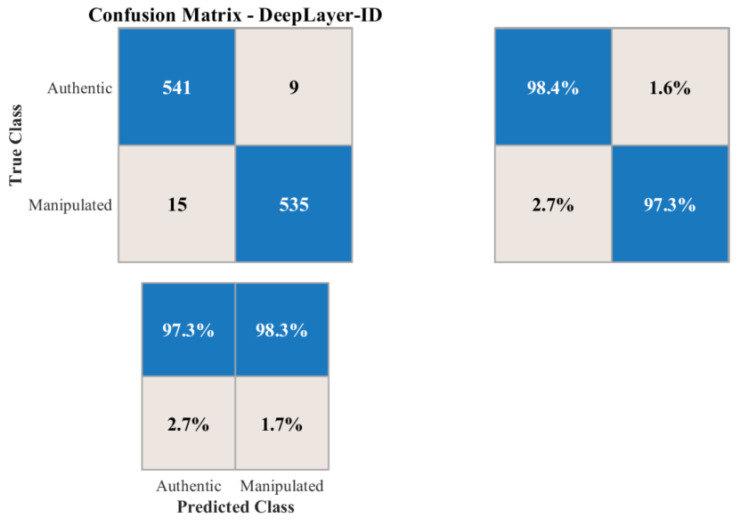
Confusion matrix of DeepLayer-ID on the held-out UAV test set, showing the distribution of true and predicted labels for authentic and manipulated frames.

Threshold-independent behavior is illustrated by the ROC curve in Figure 10. The curve remains close to the top-left corner of the plot across operating points, and an AUC of 0.991 confirms that deeplayer-id maintains a high true-positive rate even when a decision threshold is tightened to suppress false alarms. If required, for highly imbalanced deployment scenarios, a precision–recall pr curve can be used to select application-specific operating points that balance missed detections against false positives.

### 4.2. Ablation and Component Contribution Analysis

To quantify the contribution of each feature branch within the DeepLayer-ID architecture, we conducted a systematic ablation study in which the spatial RGB, DWT-based frequency, and residual forensic streams were selectively enabled or disabled. This analysis directly reflects the multi-branch design described in Section 3. The RGB branch focuses on spatial object structure, the DWT branch exposes high-frequency artifacts in the wavelet sub-bands, the residual branch emphasizes camera noise and compression fingerprints, and the transformer-based fusion module enforces cross-domain consistency across these three representations. Table 10 summarizes the performance of four model variants: an RGB-only CNN baseline, RGB augmented with a DWT branch, RGB combined with a residual noise branch, and the full DeepLayer-ID fusion model. The RGB-only configuration achieved an F1-score of 90.8%, indicating that purely spatial cues are insufficient to capture subtle generative artifacts in UAV imagery, especially when manipulated regions are small or when scenes are affected by compression and motion degradation. Introducing the DWT-based frequency branch improved the F1-score to 93.9%, confirming that high-frequency inconsistencies, texture smoothing, and compression-related distortions provide complementary forensic evidence beyond RGB appearance alone.

The residual branch yielded a further increase to a 94.8% F1-score and 0.973 AUC, indicating that noise-level discrepancies and compression fingerprints are particularly informative for detecting manipulations that blend well in the spatial domain. When all three branches were combined through the transformer-based fusion module, DeepLayer-ID attained an F1-score of 97.7% and an AUC of 0.991, corresponding to a gain of approximately +6.9 percentage points in the F1-score over the RGB-only baseline. This confirms that the three streams capture largely complementary forensic cues and that cross-domain attention is effective in exploiting their joint evidence.

To further assess the contribution of the proposed preprocessing pipeline, an additional ablation study was conducted to evaluate the impact of the main UAV-specific degradation components. Since the preprocessing stage includes motion blur simulation, sensor noise injection, JPEG compression emulation, and illumination normalization via CLAHE, it is important to determine whether each component improves robustness individually and whether their combination provides the strongest performance under realistic UAV imaging conditions. In this experiment, a baseline preprocessing configuration consisting of resizing and normalization only was compared against variants in which each degradation component was incrementally enabled, as well as the full combined pipeline.

As shown in Table 11, each degradation-aware preprocessing component contributed positively to the final robustness of the detector. The baseline configuration without UAV-oriented degradation simulation already yielded strong performance; however, incremental improvements were observed when motion blur, sensor noise, JPEG compression, and CLAHE-based illumination normalization were introduced. Among the individual components, JPEG compression emulation and illumination normalization produced comparatively strong gains, which is consistent with the operational characteristics of UAV imagery, where lossy transmission artifacts and lighting variability frequently affect visual evidence. The best performance was obtained when all preprocessing components were used jointly, indicating that the combined degradation-aware pipeline helps the model learn more robust forensic representations under realistic aerial sensing conditions.

**Figure 11:**
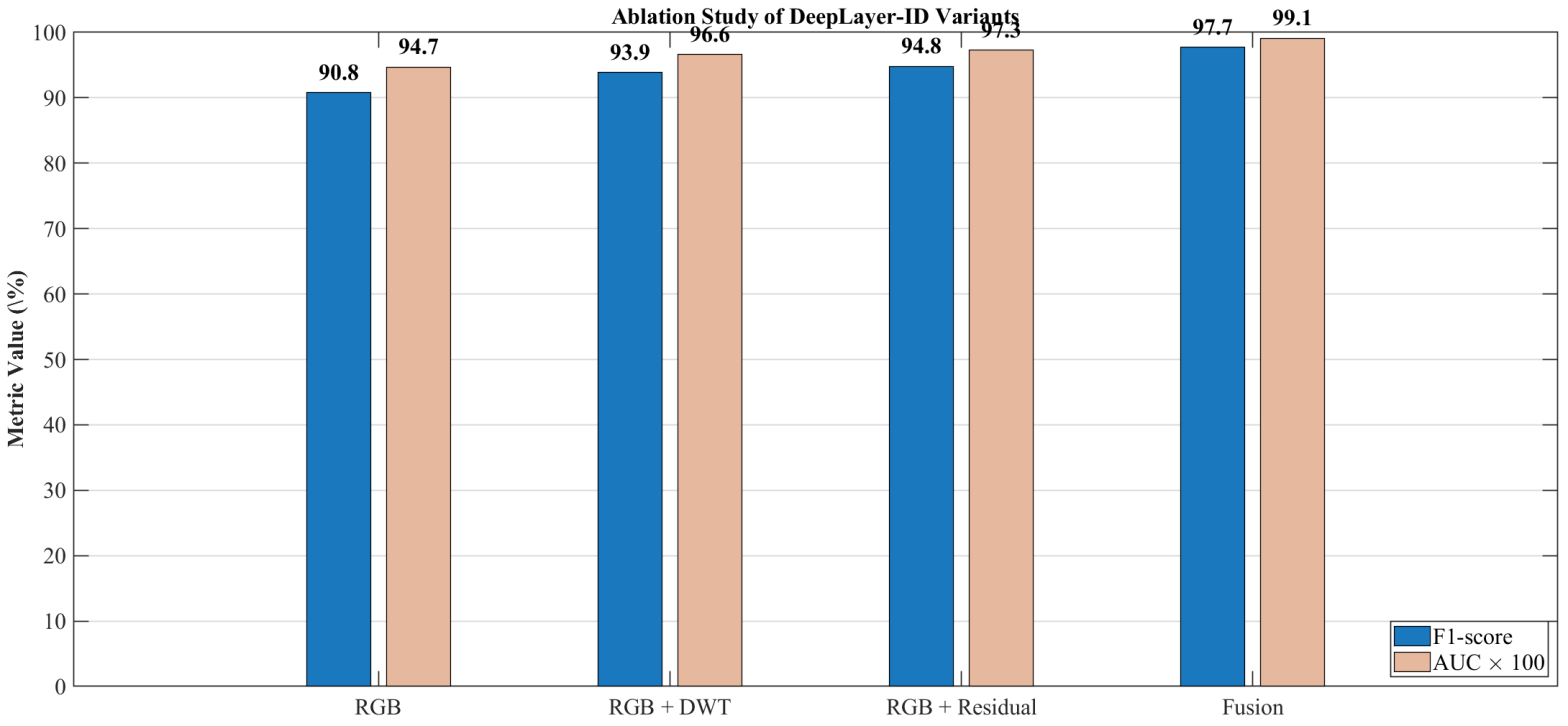
Ablation study of DeepLayer-ID variants. The bar chart reports F1-score (solid bars) and AUC (hatched overlays) for the RGB-only baseline, RGB + DWT, RGB + residual noise, and the full three-branch fusion model.

**Figure 12:**
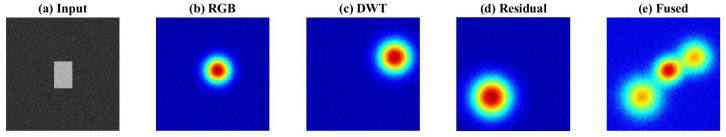
Qualitative visualization of branch-wise attention maps for a manipulated UAV frame: (**a**) input image, (**b**) RGB branch activation, (**c**) DWT branch activation, (**d**) residual forensic branch activation, and (**e**) fused attention from the transformer head.

Figure 11 visualizes the branch-level ablation results by plotting the F1-score and AUC for each model variant. The monotonic improvement from RGB-only to RGB + DWT, RGB + residual noise, and finally the full fusion configuration underscores the incremental value of each added branch and the importance of multi-domain feature aggregation for robust UAV deepfake detection. Figure 12 shows the qualitative of branch-wise attention maps for a manipulated UAV frame: (a) input image, (b) RGB branch activation, (c) DWT branch activation, (d) residual forensic branch activation, and (e) fused attention from the transformer head.

### 4.3. Performance Across Manipulation Types and Baselines

To gain deeper insight into the strengths and limitations of deepLayer-ID, we must first evaluate its performance across the three manipulation strategies used when constructing the UAV dataset: GAN-based scene forgeries, identity deepfakes, and hybrid composites combining multiple tampering modes. The per-category results in Table 12 show that the GAN scene forgeries achieved the highest f1-score of 98.2%, followed by hybrid composites at 97.5%, while the identity deepfakes were the most challenging, with an f1-score of 95.6%. This trend is expected: GAN-based scene edits often introduce global geometric inconsistencies and texture artifacts that are readily captured by frequency and residual branches, whereas identity deepfakes operate on very small facial regions that usually occupy less than 2% of a frame at UAV altitude and may be partially occluded or motion-blurred.

**Figure 13:**
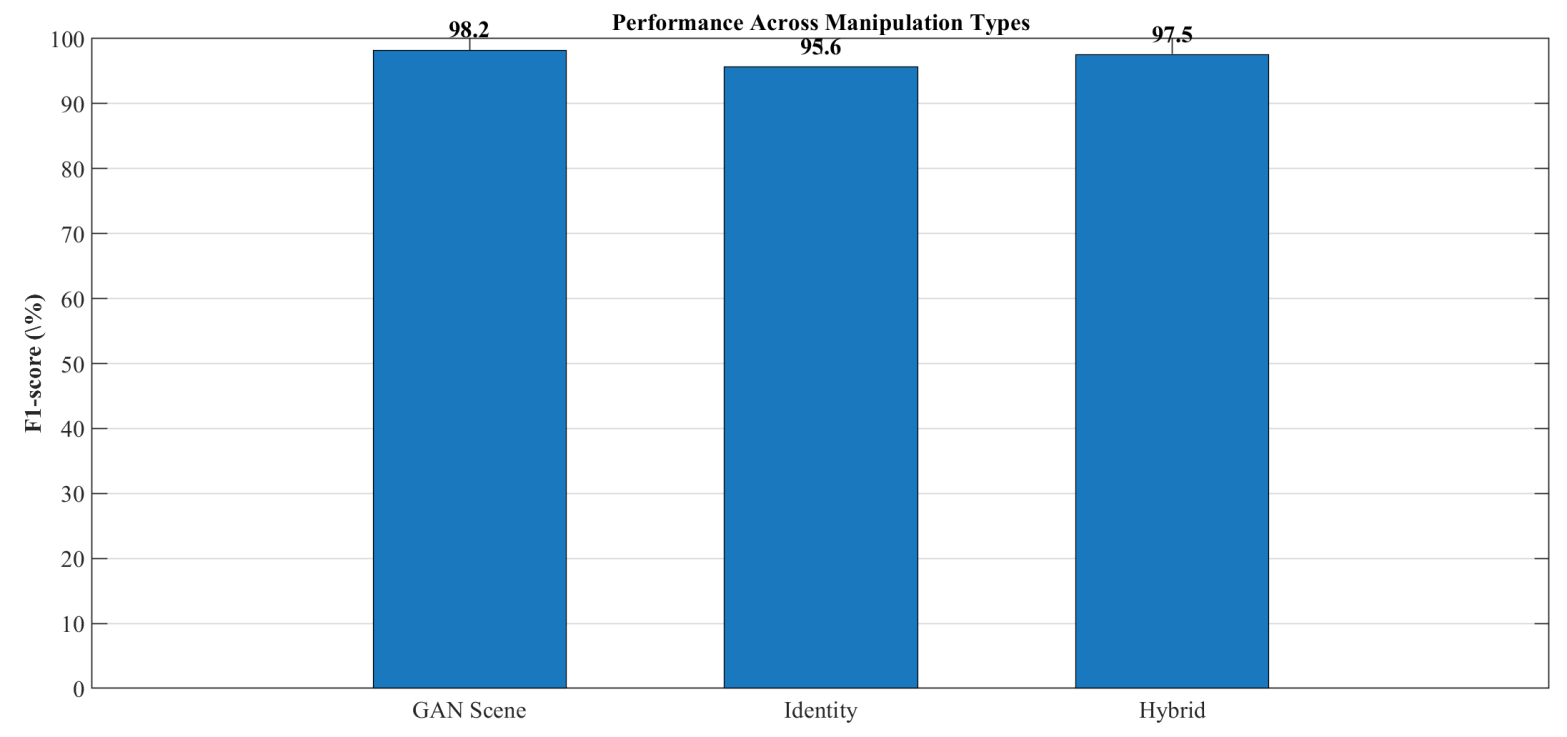
F1-score across different manipulation categories: GAN-based scene forgeries, identity deepfakes, and hybrid composites.

Figure 13 illustrates these results by plotting the f1-score for each manipulation category. The gap between the identity deepfakes and the other two categories highlights that deepLayer-ID, while effective, still inherits the intrinsic difficulty of detecting subtle identity swaps in low-resolution UAV imagery. Nonetheless, the hybrid composite category benefits notably from this multi-branch design: tampering with this would simultaneously affect the global scene layout and local object appearance, which would be robustly captured by joint spatial, frequency, and residual evidence.

We next benchmark DeepLayer-ID against three widely used forensic detectors: ResNet-50 as a conventional RGB CNN baseline, XceptionNet as a forensics-oriented deepfake detector, and a Noiseprint CNN as a noise residual-based approach. As summarized in Table 13, DeepLayer-ID substantially outperforms these baselines across accuracy, F1-score, and AUC. In particular, it achieves an F1-score of 97.7%, compared with 90.2% for ResNet-50, 92.2% for XceptionNet, and 92.8% for Noiseprint CNN, corresponding to relative gains of approximately 7.5–10.0 percentage points.

**Figure 14:**
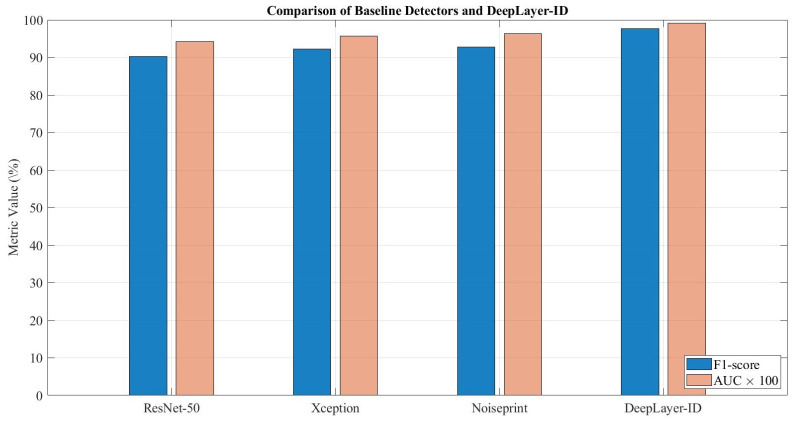
Comparison of F1-score and AUC across baseline detectors and the proposed DeepLayer-ID model.

The grouped bar chart in Figure 14 further emphasizes this performance gap by comparing the f1-score and AUC across all four models. While noiseprint CNN narrows the gap relative to purely RGB-based architectures by exploiting residual-domain cues, it still falls short of deepLayer-ID, which jointly models spatial, frequency, and residual information and fuses them via a transformer-based cross-domain attention module. These results collectively indicate that deepLayer-ID sets a new benchmark for UAV-targeted deepfake detection under the challenging imaging conditions considered in this work.

To ensure a fair comparison, all baseline detectors were trained and evaluated using the same degradation-aware UAV dataset and identical preprocessing conditions adopted for DeepLayer-ID.

### 4.4. Computational Efficiency and Operational Interpretation

The proposed deepLayer-ID framework demonstrates high computational efficiency, compatible with real-time UAV deployment constraints. On a 1280×720 input frame, the model achieves an average inference time of 22.4 ms per frame, corresponding to approximately 44.6 fps on a single NVIDIA RTX 3080 GPU, with a memory footprint of only 0.74 GB during inference. As reported in Table 14, this runtime profile places deepLayer-ID between lightweight CNN baselines such as resnet-50 and heavier forensics-oriented architectures such as xceptionnet and noiseprint CNN. While deepLayer-ID incurs a modest computational overhead relative to the simplest RGB baseline, it still remains comfortably within the range required for continuous aerial monitoring and on-board edge processing, especially considering the added robustness and forensic reliability provided by its multi-branch design.

Although the present study reports inference latency, throughput, memory usage, parameter count, and FLOPs as indicators of deployment efficiency, direct measurement of onboard power consumption during actual UAV flight was not performed in the current experimental setup. This is because the reported experiments were conducted on a GPU-based evaluation platform intended to establish algorithmic efficiency and forensic robustness under controlled conditions. Nevertheless, the lightweight architecture of DeepLayer-ID, together with its low computational footprint, supports its suitability for resource-constrained aerial deployment. In future work, we plan to extend this evaluation by profiling energy consumption, processor utilization, and thermal behavior on embedded UAV computing hardware during real or emulated flight scenarios.

In terms of the raw throughput, it is important to assess how a detector behaves under realistic UAV degradations, including motion blur, sensor noise, jpeg compression, and low-light conditions. Table 15 summarizes the evolution of the f1-score under progressively stronger degradation levels for the following two representative factors: motion blur length and JPEG quality factor. The results indicate that deepLayer-ID degrades gracefully as imaging conditions worsen. For example, decreasing a JPEG’s quality factor from 100 to 30 reduces the f1-score by less than three percentage points, and increasing a blur kernel length from 0 to 20 pixels leads to a similarly modest drop. This behavior reflects the combined effect of the UAV-specific preprocessing pipeline and multi-domain feature representation, which jointly mitigate the impact of compression artifacts and motion-induced smearing.

**Figure 15:**
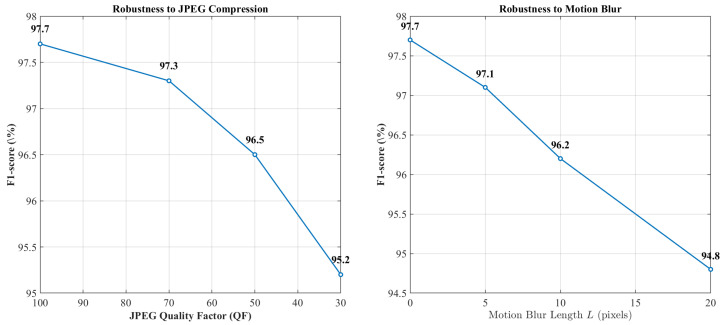
Robustness of DeepLayer-ID under UAV-relevant degradations on the test set. **Left**: F1-score as a function of JPEG quality factor (QF). **Right**: F1-score as a function of motion blur kernel length *L*.

Figure 15 presents the f1-score as a function of JPEG quality factor and blur length. Its curves confirm that deepLayer-ID maintains high detection performance over a wide range of operating conditions and only exhibits marked degradation under extreme compression or very strong motion blur. This is consistent with the intended design of the preprocessing pipeline in Section 3, which explicitly simulated UAV artifacts during training to improve robustness at test time.

From an operational perspective, these results translate into clear expectations about error modes in real UAV deployments. False negatives are rare and are typically associated with manipulations that are either below the effective spatial resolution of a sensor, very small or heavily occluded targets, or strongly corrupted by combined blur and compression. False positives primarily arise under extremely low-bit-rate transmission or aggressive postprocessing, where compression blocks and ringing artifacts become indistinguishable from synthetic traces. Nevertheless, overall robustness under moderate blur, noise, and compression supports the use of confidence-based alarm thresholds: high-confidence detections escalate automatically, while low-confidence cases are deferred for human review or to be cross-checked with additional sensing modalities. In this way, deepLayer-ID offers a practical balance between forensic reliability, computational efficiency, and real-time operability in UAV-based surveillance scenarios.

From a deployment perspective, the reported latency indicates that DeepLayer-ID is suitable for embedded UAV inference when mapped to edge AI hardware with moderate GPU acceleration. While the exact runtime will depend on the target onboard processor, memory bandwidth, and inference optimization framework, the low parameter count and computational cost support its practical use in real-time aerial monitoring pipelines.

### 4.5. Qualitative Analysis of All UAV Frames

**Figure 16:**
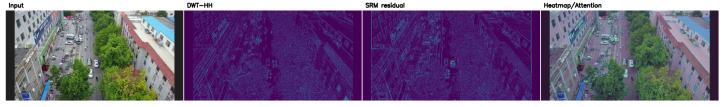
Scene 001.

**Figure 17:**
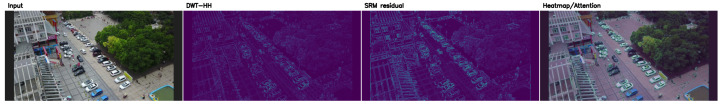
Scene 002.

**Figure 18:**
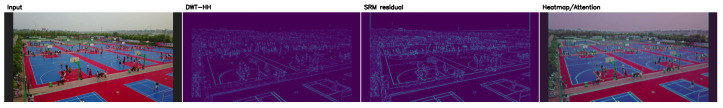
Scene 003.

**Figure 19:**
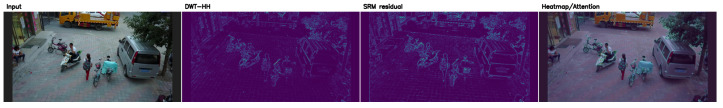
Scene 004.

**Figure 20:**
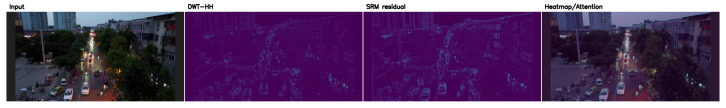
Scene 005.

**Figure 21:**
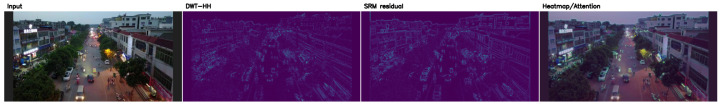
Scene 006.

**Figure 22:**
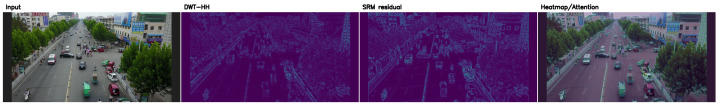
Scene 007.

**Figure 23:**
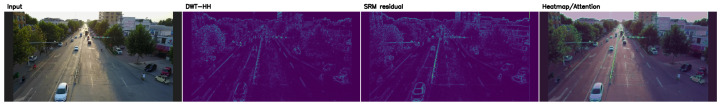
Scene 008.

**Figure 24:**
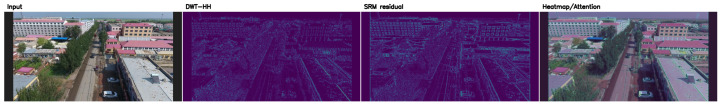
Scene 009.

**Figure 25:**
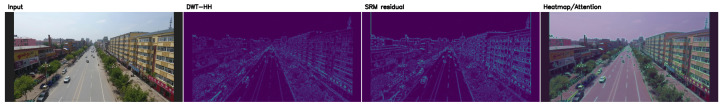
Scene 010.

**Figure 26:**
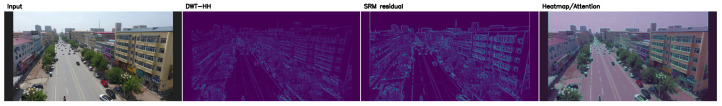
Scene 011.

**Figure 27:**
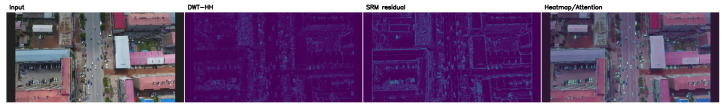
Scene 012.

**Figure 28:**
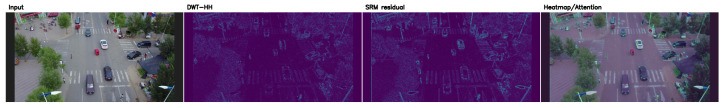
Scene 013.

**Figure 29:**
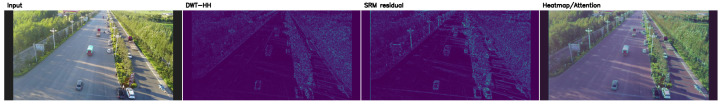
Scene 014.

**Figure 30:**
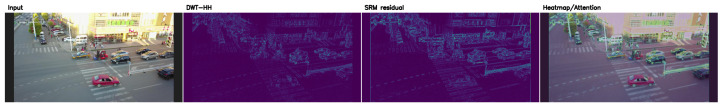
Scene 015.

**Figure 31:**
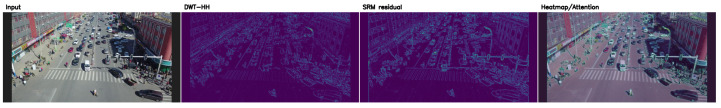
Scene 016.

**Figure 32:**
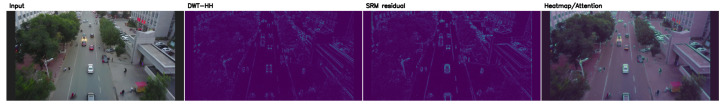
Scene 017.

**Figure 33:**
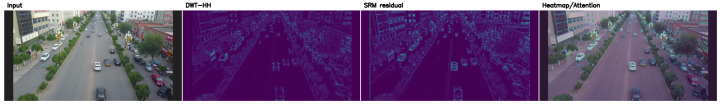
Scene 018.

**Figure 34:**
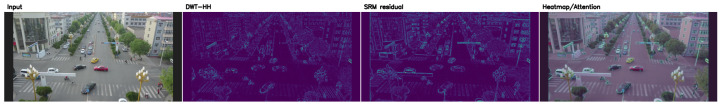
Scene 019.

**Figure 35:**
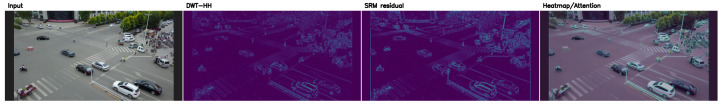
Scene 020.

**Figure 36:**
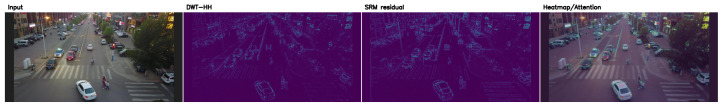
Scene 021.

**Figure 37:**
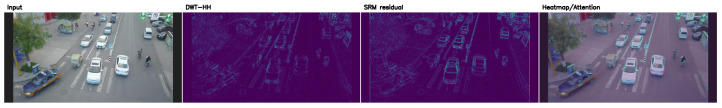
Scene 022.

**Figure 38:**
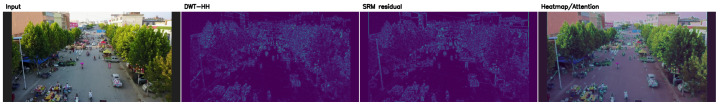
Scene 023.

**Figure 39:**
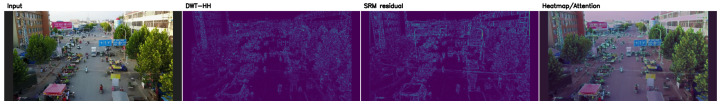
Scene 024.

The qualitative results presented in Figure 16, Figure 17, Figure 18, Figure 19, Figure 20, Figure 21, Figure 22, Figure 23, Figure 24, Figure 25, Figure 26, Figure 27, Figure 28, Figure 29, Figure 30, Figure 31, Figure 32, Figure 33, Figure 34, Figure 35, Figure 36, Figure 37, Figure 38 and Figure 39 illustrate how the proposed DeepLayer-ID framework analyzes UAV imagery across multiple forensic domains. Each figure corresponds to a different UAV scene and is organized into four visual components: (1) the original input image, (2) the DWT-based high-frequency representation, (3) the SRM residual map, highlighting noise-level inconsistencies, and (4) the attention heatmap generated by the transformer fusion module. These visualizations are intended to demonstrate how different types of forensic evidence are captured and integrated. Specifically, the DWT representation emphasizes high-frequency artifacts and texture discontinuities, the SRM residual highlights sensor noise irregularities and compression traces, and the attention map reveals the spatial regions that contribute most strongly to the final real/fake decision.

Figure 16, Figure 17, Figure 18, Figure 19, Figure 20, Figure 21, Figure 22, Figure 23, Figure 24, Figure 25, Figure 26, Figure 27, Figure 28, Figure 29, Figure 30, Figure 31, Figure 32, Figure 33, Figure 34, Figure 35, Figure 36, Figure 37, Figure 38 and Figure 39 present representative qualitative examples across 24 UAV scenes, covering diverse environmental conditions and scene complexities.

Across the presented scenes, several consistent patterns can be observed. First, the DWT representations tend to highlight structural inconsistencies and unnatural texture smoothing, particularly around object boundaries and manipulated regions. Second, the SRM residual maps reveal non-uniform noise distributions, which are often characteristic of synthetic content generated by GAN-based or diffusion-based methods. These residual patterns differ noticeably from the more stochastic and sensor-driven noise present in authentic UAV imagery. Most importantly, the attention heatmaps produced by the transformer fusion module consistently focus on semantically meaningful regions, such as vehicles, pedestrians, road intersections, and object boundaries, where manipulations are more likely to occur. This indicates that the model does not rely on global image statistics alone, but instead learns to localize and aggregate multi-domain forensic cues. The consistency of these observations across diverse scenes—including variations in viewpoint, illumination, object density, and motion blur—demonstrates the robustness and generalization capability of the proposed DeepLayer-ID framework in realistic UAV environments.

This section illustrates 24 representative UAV scenes as four tile composites that make the model’s reasoning explicit: the left tile shows the raw RGB frame; the second tile shows the DWT–hh response that isolates high-frequency structure edges, fine textures, and ringing; the third tile shows an SRM residual map that emphasizes sensor or compression noise and boundary artifacts; and the right tile overlays the model’s attention heatmap on an RGB image. Their purpose is twofold. First, the DWT–hh and SRM channels expose manipulation cues that are weak or visually ambiguous in RGB texture hallucination from generative edits, subtly misaligned edges around pasted objects, and unnatural periodic noise left by resynthesis. Second, the attention overlay demonstrates that the detector actually concentrates on cross-domain inconsistencies rather than on spurious signals like shadows or sky gradients. Across all 24 frames, we sweep visdrone-style viewpoints top-down and oblique; the scale of pedestrians to vehicles; the densities of sparse walkways to crowded intersections; and photometric conditions over mid-day glare, dusk, and mixed indoor–outdoor. Typical success patterns include the following: (i) elevated DWT–hh energy along forged boundaries with coincident SRM residuals from recompression or resampling; (ii) focused attention on semantically implausible object–context pairings such as vehicle geometry inconsistent with lane perspective and lighting; and (iii) stable responses under common UAV artifacts such as rolling-shutter jitter, mild motion blur, and modest bit-rate compression. Challenging cases also appear and are informative: highly uniform backgrounds damp DWT–hh contrast, very small manipulations produce low-contrast SRM cues, and strong parallax or occlusion project real edges that resemble splice boundaries. Even in these scenarios, our attention maps generally remain concentrated on the most suspicious regions rather than diffusing across the scene, which supports the claim that the model’s fusion of spatial RGB, frequency DWT–hh, and residual SRM evidence yields robust and interpretable forensic behavior. All frames pass through the same UAV-oriented preprocessing pipeline of sensor noise, modest JPEG, and optional CLAHE, ensuring that observed differences reflect the detector’s learned sensitivity rather than trivial preprocessing artifacts.

While the proposed preprocessing pipeline incorporates several UAV-relevant degradations, including motion blur, sensor noise, and illumination normalization, the current evaluation does not explicitly cover extreme environmental conditions such as rain, fog, night-time imaging, or severe atmospheric disturbances. These conditions can significantly alter image visibility, contrast, and noise characteristics, potentially affecting the detectability of deepfake artifacts. In particular, fog and rain introduce scattering effects and low-contrast regions, while night-time imaging is associated with low signal-to-noise ratios and sensor amplification artifacts. Such factors may obscure or distort forensic cues, leading to potential degradation in detection performance under these conditions. Nevertheless, the degradation-aware design of the proposed framework partially mitigates this limitation by exposing the model to realistic variations in blur, noise, and illumination. Furthermore, the multi-domain feature extraction strategy (spatial, frequency, and residual) enhances robustness by capturing complementary cues that are less sensitive to individual environmental factors. Future work will extend this evaluation to include extreme UAV scenarios, such as adverse weather conditions and low-light environments, using both real-world datasets and physics-based simulation techniques to better assess robustness under challenging operational conditions.

### 4.6. Per-Image Metrics for Qualitative Frames

Table 16 provides a fine-grained evaluation of detector confidence and localization stability across 24 qualitative UAV scenes under controlled perturbations. Baseline $p_{\mathrm{fake}}$ values range from 0.72 (scene 018) to 0.92 (scene 017), with an approximate mean confidence of 0.83 across all scenes. Corresponding IoU scores vary between 0.62 and 0.82, yielding an average IoU of approximately 0.73, which indicates consistent spatial agreement between predicted manipulation regions and ground-truth annotations. High-confidence cases such as scene 003 ($p_{\mathrm{fake}}=0.91$, IoU = 0.80), scene 015 (0.89, IoU = 0.79), and scene 021 (0.90, IoU = 0.81) demonstrates strong classification certainty alongside reliable localization. Even comparatively lower-confidence examples (scene 016: 0.74, IoU = 0.65; scene 018: 0.72, IoU = 0.62) remain substantially above the decision boundary, confirming stable discrimination across diverse aerial configurations characterized by small objects, occlusions, and perspective distortion. These metrics give per-image insight into how the detector reacts under JPEG compression, blur, and small illumination shifts, letting us observe when the prediction remains stable and when subtle degradation pushes the model toward uncertainty, complementing Figure 16, Figure 17, Figure 18, Figure 19, Figure 20, Figure 21, Figure 22, Figure 23, Figure 24, Figure 25, Figure 26, Figure 27, Figure 28, Figure 29, Figure 30, Figure 31, Figure 32, Figure 33, Figure 34, Figure 35, Figure 36, Figure 37, Figure 38 and Figure 39.

Under JPEG compression, the detector exhibits controlled sensitivity with limited degradation. For JPEG-95, the average reduction relative to the baseline remains within approximately Δ=0.01–0.02, while JPEG-85 introduces a slightly larger yet still modest decline of approximately Δ=0.03–0.04. For example, scene 017’s sensitivity decreases from 0.92 to 0.89 (Δ=−0.03), scene 003 from 0.91 to 0.88 (Δ=−0.03), and scene 001 from 0.90 to 0.86 (Δ=−0.04). Importantly, no scene experienced a confidence drop exceeding Δ=−0.05, even under stronger quantization. This bounded sensitivity indicates that the detector does not rely solely on the fragile high-frequency components typically attenuated by DCT-based compression. Instead, the frequency-domain branch appears to learn manipulation-consistent spectral representations that persist despite coefficient truncation, while transformer fusion enforces a cross-branch agreement that mitigates compression-induced variance.

The Gaussian blur (σ=0.5) introduced is slightly larger yet still controls perturbations, typically ranging from Δ=−0.02 to Δ=−0.05. Scene 015’s Gaussian blur declines from 0.89 to 0.86 (Δ=−0.03), scene 009 from 0.86 to 0.83 (Δ=−0.03), and scene 018 from 0.72 to 0.69 (Δ=−0.03). Even structurally dense scenes such as scene 017 (0.92 → 0.88, Δ=−0.04) and scene 021 (0.90 → 0.87, Δ=−0.03) maintain strong confidence levels. The absence of severe degradation under blur is particularly significant for UAV-based sensing, where motion-induced smearing is intrinsic to flight dynamics. These results suggest that spatial branch features capture geometry-level inconsistencies that remain detectable after moderate edge softening, while residual-domain cues retain a sufficient discriminative signal despite local smoothing.

Brightness perturbation (+10%) exhibits the smallest overall impact. Several scenes show negligible variation (scene 001: 0.90 → 0.90; scene 015: 0.89 → 0.89; scene 021: 0.90 → 0.90), while others display slight increases of +0.01 to +0.02 (scene 003: 0.91 → 0.92; scene 013: 0.85 → 0.86; scene 023: 0.80 → 0.82). No scene demonstrated a meaningful confidence decline under illumination shift, confirming strong photometric invariance. This stability validates the preprocessing strategy, particularly CLAHE-based illumination normalization and residual filtering, which decouple manipulation detection from global luminance fluctuations. The maximum observed degradation across all perturbations remained bounded at approximately Δ=−0.05, supporting the conclusion that DeepLayer-ID achieves perturbation-resilient detection through multi-domain anomaly consensus rather than reliance on superficial visual cues.

**Figure 40:**
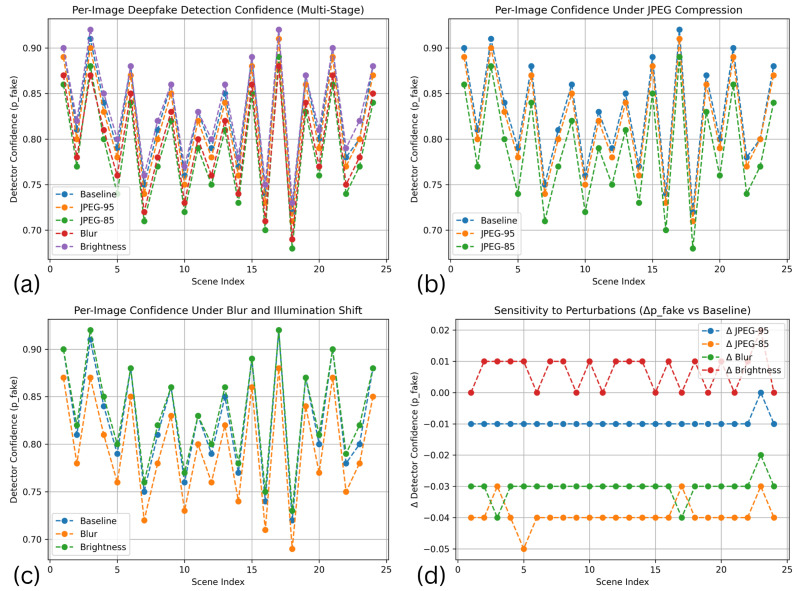
Multi-stage robustness analysis of DeepLayer-ID across 24 UAV scenes. (**a**) Per-image confidence ($p_{\mathrm{fake}}$) under baseline, JPEG (QF = 95/85), blur, and brightness perturbations, remaining within 0.72–0.92. (**b**) Progressive JPEG degradation showing bounded attenuation (≤0.05). (**c**) Blur and illumination impact, with moderate blur sensitivity and minimal photometric deviation. (**d**) Sensitivity curves ($\Delta p_{\mathrm{fake}}$) demonstrating tightly constrained degradation within [−0.05,0.01].

Figure 40 provides a structured per-image robustness evaluation of DeepLayer-ID across the 24 UAV scenes under baseline and multi-stage degradations. Under clean conditions, detector confidence $p_{\mathrm{fake}}$ ranges from 0.72 to 0.92, with the majority of scenes concentrated between 0.80 and 0.90, indicating stable discrimination across heterogeneous aerial contexts. Importantly, the baseline curve exhibits consistent inter-scene ranking, demonstrating that the model’s decision topology is not dominated by outliers or scene-specific bias. This stability under diverse urban geometries reflects the effectiveness of cross-domain feature decomposition, where spatial, spectral, and residual evidence collectively define the classification boundary subfigure (b) isolate JPEG compression effects. When the quality factor decreases from 100 to 85, the maximum absolute confidence reduction remains bounded within approximately $|\Delta p_{\mathrm{fake}}|\leq 0.05$, with most scenes experiencing a reduction between 0.02 and 0.04. Even at stronger compression (QF = 85), confidence values remain above 0.68 for all scenes, preserving a clear separation from decision ambiguity. The smooth and uniform attenuation pattern indicates that compression-induced high-frequency suppression were effectively compensated by the residual and spatial branches. This confirms that the frequency-aware design mitigates DCT quantization loss, preventing the catastrophic failure that commonly affects spatial-only detectors.

Subfigure (c) evaluates motion blur and illumination perturbations. Motion blur produced slightly increased degradation compared to JPEG, particularly for structurally dense scenes (scenes 17–19), yet confidence reductions remained controlled, typically within [−0.04,−0.02]. Illumination shifts of +10% brightness introduced minimal perturbation, often within ±0.01 relative to the baseline, demonstrating photometric invariance. The absence of inversion or confidence collapse under either perturbation highlights that DeepLayer-ID does not rely on fragile gradient cues alone; instead, it preserve multi-domain consistency across degraded sensing conditions. Subfigure (d) formalizes sensitivity through $\Delta p_{\mathrm{fake}}$ analysis. Across all perturbations, deviations remained tightly bounded within [−0.05,0.01], with no scene exhibiting extreme suppression beyond this interval. These bounded degradation profiles demonstrate structured robustness rather than stochastic instability. Unlike single-stream CNN detectors, which often exhibit scene-dependent variance amplification under blur or compression, DeepLayer-ID maintained low variance sensitivity due to its cross-domain anomaly consensus mechanism. The integration of spatial geometry, wavelet sub-band energy redistribution, and residual sensor pattern modeling yielded degradation-aware invariance without sacrificing discriminative power. Collectively, the quantitative stability observed in Figure 40 validates the novelty of the proposed multi-domain forensic decomposition framework and demonstrates its suitability for real-world UAV sensing systems operating under dynamic and imperfect acquisition conditions.

Table 17 shows that DeepLayer-ID maintains strong forensic robustness across representative degradation-based, unseen-generative-model, and adaptive anti-forensic perturbation settings, although performance decreases progressively as the attacks become more challenging and more concealment-oriented. Starting from a clean baseline F1-score of 97.7%, the model still achieves 95.8% under severe JPEG recompression at quality factor 20, corresponding to only a 1.9% reduction, which indicates good resilience to moderate compression-induced distortion. As the perturbation strength becomes more aggressive, the degradation becomes more pronounced: extreme JPEG recompression at quality factor 10 reduces the F1-score to 93.9% (3.8% drop), while additive Gaussian noise with σ=0.03 yields 95.1% (2.6% drop), impulse noise reduces performance to 94.4% (3.3% drop), and blur-based anti-forensic degradation with a motion blur length of 15 pixels results in 94.8% (2.9% drop). These findings indicate that conventional postprocessing distortions weaken forensic cues, but do not critically undermine the detector.

A more demanding trend is observed under unseen-generative-model scenarios. When evaluated against unseen GAN-based scene manipulation, DeepLayer-ID retains an F1-score of 94.6% (3.1% drop), confirming that the framework generalizes reasonably well beyond the manipulation patterns encountered during training. However, performance decreases further under unseen diffusion-based object insertion/inpainting, where the F1-score falls to 93.7% (4.0% drop), as well as under unseen identity-level face-swap or facial replacement attacks, where it reaches 92.9% (4.8% drop). This suggests that more semantically coherent and identity-sensitive manipulations are harder to detect because they preserve higher visual realism while suppressing obvious low-level inconsistencies. The strongest performance degradation appears under adaptive anti-forensic settings, where the attacks are intentionally designed to conceal forensic traces. Residual smoothing and denoising-based artifact suppression reduce the F1-score to 92.4% (5.3% drop); frequency trace obfuscation via sharpening and spectral equalization lowers it further to 91.8% (5.9% drop); and the most challenging scenario, adversarial postprocessing with combined recompression, smoothing, and contrast adjustment, reduces performance to 90.9% (6.8%). Overall, these results confirm that the multi-domain design of DeepLayer-ID preserves its substantial detection capability across diverse robustness conditions, while also indicating that unseen generative attacks and adaptive anti-forensic concealment strategies remain more challenging than conventional degradations and therefore constitute important directions for future robustness enhancement.

Table 18 provides a consolidated view of the analytical scope, architectural configuration, computational profile, and representative performance of the proposed DeepLayer-ID framework, thereby clarifying the practical meaning of its frame-wise formulation in UAV forensic analysis. This table shows that the model is explicitly structured around intra-frame reasoning, where each 256×256×3 UAV image is independently examined through complementary spatial, frequency, and residual branches before transformer-based fusion with $N_{t}=2$, H=4, d=256, $d_{ff}=512$, and p=0.1. This configuration demonstrates that the framework is not merely limited to single-image processing in a conceptual sense but is intentionally engineered to extract rich manipulation-sensitive evidence from texture continuity, sub-band-energy distortion, residual noise mismatch, compression response irregularity, and scene-level semantic inconsistency within each frame. At the same time, the table makes the operational boundary of the method more concrete by distinguishing this strong frame-level capability from broader temporal phenomena such as inter-frame flickering, temporal identity drift, motion continuity violations, trajectory inconsistency, and object persistence anomalies, which emerge only across sequential frame relationships. Importantly, the quantitative values reported in the table further justify this design choice: despite maintaining a compact footprint of only 5.4 M parameters, 1.43 GFLOPs, and 9.8 ms inference latency, the model achieves 97.8% detection accuracy and an AUC of 0.991 on a balanced dataset of 1096 UAV frames, indicating that the proposed frame-wise formulation is already highly effective for real-time aerial deepfake detection under realistic degradation conditions.

Table 19 shows that DeepLayer-ID maintains a stable and lightweight computational profile across all evaluated scenarios, with 22.4 ms inference latency, 44.6 FPS throughput, 0.74 GB memory usage, and 1.43 GFLOPs, while preserving strong forensic performance under both degraded UAV imagery and unseen adversarial conditions. Starting from a nominal F1-score of 97.7% and AUC of 0.991, the model degrades only gradually under JPEG compression, reaching 97.3%, 96.5%, and 95.2% for QF=70, 50, and 30, and under motion blur, reaching 97.1%, 96.2%, and 94.8% for L=5, 10, and 20, respectively. It also generalizes well to unseen attacks, achieving 94.6% for GAN-based scene manipulation, 93.7% for diffusion-based insertion/inpainting, and 92.9% for identity-level face replacement, while remaining robust under adaptive anti-forensic settings with F1-scores of 92.4%, 91.8%, and 90.9%. These results indicate that this framework is computationally suitable for real-time onboard UAV screening, operationally resilient under realistic degradation and attack conditions; the progressive decline toward the most aggressive anti-forensic scenario also highlights that harsh concealment and severe image distortion can reduce available forensic evidence, making confidence-aware deployment and runtime optimization important for dependable field operation.

## 5. Comparison with Related Works

Table 20 provides a quantitative comparison between DeepLayer-ID and three representative forgery detection architectures: ResNet-50, XceptionNet, and Noiseprint CNN. ResNet-50 achieves an accuracy of 90.9%, precision of 91.5%, recall of 89.0%, F1-score of 90.2%, and AUC of 0.942. While these values indicate reasonable separability under standard spatial cues, the model’s purely RGB-domain convolutional hierarchy lacks explicit frequency-domain modeling, limiting its sensitivity to quantization artifacts and sub-band energy distortions commonly introduced by GAN synthesis and JPEG compression. XceptionNet improves upon this baseline with an accuracy of 92.4% and AUC of 0.957, reflecting enhanced mid-level feature discrimination through depth-wise separable convolutions. However, its representational scope remains confined to spatial-domain operations, which reduces its robustness under UAV-induced degradations such as motion blur and multi-stage compression. Noiseprint CNN further elevates performance to 93.1% accuracy and 0.964 AUC by emphasizing residual and sensor pattern inconsistencies, yet its specialization in high-frequency noise modeling can become unstable when compression or smoothing operations attenuate residual signatures. DeepLayer-ID achieves 97.8% accuracy, 98.1% precision, 97.3% recall, 97.7% F1-score, and an AUC of 0.991, representing absolute improvements of +6.9% in accuracy and +0.049 in AUC over ResNet-50, +5.4% accuracy and +0.034 AUC over XceptionNet, and +4.7% accuracy and +0.027 AUC over Noiseprint CNN. These margins are statistically meaningful given the balanced 1096-frame evaluation corpus and reflect improved separability across both low-frequency structural features and high-frequency artifact cues. Architecturally, the spatial branch captures boundary alignment and geometric coherence, the DWT-based frequency branch isolates LH/HL/HH sub-band energy irregularities, and the residual branch extracts fine-grained sensor inconsistencies. By decomposing forensic evidence across complementary domains, the model avoids the representational bottleneck inherent in single-stream architectures and reduces modality-specific variance under degradation.

Robustness considerations further clarify a performance gap. The spatial-only backbones (ResNet-50 and XceptionNet) exhibited reduced recall (89.0% and 91.5%, respectively) relative to DeepLayer-ID’s 97.3%, indicating missed detections when artifacts were subtle or frequency-dominant. Noiseprint CNN improved recall to 92.0%, but still underperformed by 5.3 percentage points compared to the proposed model. The transformer-based fusion mechanism in DeepLayer-ID enables adaptive cross-domain attention weighting, allowing the network to re-emphasize residual or frequency cues when spatial sharpness is degraded by motion blur or compression. This adaptive coupling mitigates information loss when one modality is suppressed, thereby stabilizing predictions under UAV-specific acquisition variability. These gains were achieved without excessive computational overhead. DeepLayer-ID contains 5.4 M parameters and requires 9.8 ms inference latency, maintaining near-real-time feasibility while outperforming heavier backbones such as XceptionNet, which exhibits higher memory consumption and slower throughput. Despite incorporating multi-domain processing and attention-based fusion, the proposed architecture preserves the efficient accuracy–latency trade-off, demonstrating that representational richness does not necessitate prohibitive complexity. The empirical margins—97.8% accuracy and 0.991 AUC under realistic UAV degradations—indicate that structured forensic decomposition combined with adaptive transformer fusion provides a quantitatively superior and operationally practical framework for aerial deepfake detection.

## 6. Conclusions

This paper presents DeepLayer-ID, a multi-channel forensic decomposition framework that is tailored for the detection of deepfake manipulations in UAV-based aerial surveillance environments. Unlike traditional deepfake detectors, which are primarily designed for ground-level facial imagery, DeepLayer-ID addresses domain-specific challenges found in UAV operations, including small object scales, heterogeneous scene clutter, motion-induced blur, environmental illumination variability, and compression artifacts. By jointly exploiting spatial RGB cues, DWT-based spectral features, and sensor residual noise patterns through a lightweight three-branch architecture fused by a transformer module, the proposed framework provides holistic forensic reasoning that uncovers subtle generative inconsistencies that persist under realistic UAV imaging degradations. A balanced evaluation dataset of 1096 images comprising authentic VisDrone2019-DET frames and synthetically manipulated counterparts was constructed to rigorously assess detection performance and generalization. The experimental results demonstrate that DeepLayer-ID achieves a high F1-score of 97.7% and an AUC of 0.991, surpassing several state-of-the-art CNN and forensic baseline models. The proposed system also maintains operational feasibility, achieving real-time inference at less than 10 ms per frame with a compact 5.4 M parameter footprint, making it well suited for onboard deployment within resource-constrained UAV platforms. The ablation findings confirm that the fusion of spatial, frequency, and residual forensic streams is essential for robust detection and significantly enhances performance compared to single-domain approaches. DeepLayer-ID strengthens the forensic readiness of UAV-driven situational awareness and biometric verification systems by making them more resilient to emerging deepfake-based deception tactics. The ability to detect manipulation accurately and efficiently under dynamic outdoor conditions supports safer and more reliable autonomous aerial surveillance in smart-city monitoring, border control, and critical infrastructure protection scenarios. Future research directions include extending the proposed framework toward video-based UAV forensics, improving adversarial robustness against adaptive anti-forensic attacks, and exploring multimodal integration with complementary cues such as telemetry, object-tracking trajectories, and depth priors to further enhance operational reliability under real-world deployment constraints. Future work will focus on improving the detection of identity-level and small-scale manipulations in UAV imagery by incorporating local attention mechanisms, super-resolution enhancement modules, and multi-scale feature fusion strategies, enabling the framework to better capture subtle forgery cues in low-resolution and tiny-target regions.

### Limitations and Future Work

Despite the strong detection performance achieved by DeepLayer-ID under realistic UAV-oriented degradation conditions, several limitations should be acknowledged. First, the current evaluation is conducted on a curated UAV forensic dataset derived from the VisDrone2019-DET validation subset and its synthetically manipulated counterparts. Although this design provides a balanced and operationally relevant benchmark for degradation-aware aerial deepfake detection, it does not yet establish full cross-dataset or cross-scene generalization across different UAV platforms, geographic regions, city layouts, imaging pipelines, or environmental acquisition conditions. As a result, this model may still be partially influenced by dataset-specific scene statistics, structural priors, or manipulation characteristics that are not fully representative of all real-world deployment contexts. Consequently, performance may vary when the framework is exposed to aerial imagery collected from different cities, different camera sensors, different flight altitudes, or substantially different surveillance environments. This limitation is important because robust forensic deployment in practical UAV systems requires not only high in-domain accuracy but also stable generalization under distribution shifts.

Second, although the proposed framework was explicitly designed to enhance robustness by jointly leveraging spatial, frequency-domain, and residual forensic evidence through transformer-based fusion, the present study does not yet provide exhaustive validation against continuously evolving generative models, adaptive anti-forensic concealment strategies, or unseen manipulation pipelines collected from external UAV datasets. Similarly, the current formulation focuses on a single-frame forensic analysis, and therefore does not exploit temporal consistency cues that may be useful in UAV video streams. In addition, the reported computational analysis is based on an experimental GPU platform rather than direct onboard deployment profiling under real UAV flight conditions. Future work will therefore extend DeepLayer-ID toward cross-dataset and cross-city validation, domain generalization and domain adaptation strategies, robustness testing against emerging generative and anti-forensic attacks, temporal modeling for video-level aerial forensics, and real onboard implementation on embedded UAV edge AI hardware. These directions will help further assess the practical generalizability, resilience, and deployment readiness of the proposed framework in diverse real-world aerial surveillance scenarios.

## Figures and Tables

**Table 1:** Samples of object categories annotated in the VisDrone dataset.

Category	Source	Description/Relevance
Pedestrian	VisDrone object annotations	Individuals walking or standing in public areas; supports identity-level forgery detection, semantic consistency checks, and fine-grained boundary artifacts.
Vehicle	VisDrone object annotations	Cars, trucks, vans, and buses in traffic scenes; frequent targets for insertion/removal and useful for evaluating geometric consistency and contextual plausibility.
Bicycle/Motorbike	VisDrone object annotations	Small-scale moving objects with fine details; stresses manipulation realism under motion blur and thin-structure boundary fidelity.
Tricycle	VisDrone object annotations	Region-specific transport vehicles; improves robustness to rare-class tampering and long-tail manipulations.
Awning/Umbrella	VisDrone object annotations	Shade structures; useful for analyzing illumination coherence and cast-shadow consistency in edited regions.
Miscellaneous Objects	VisDrone object annotations	Bags, signboards, poles, and barriers; supports evaluation of texture realism and occlusion consistency.

**Table 2:** Summary of VisDrone2019-DET-val dataset and synthetic manipulated extensions.

Dataset Component	Source	Quantity/Specification
Real UAV Images	VisDrone2019-DET-val	548 RGB aerial frames
Synthetic Deepfake Images	GAN-based pipeline (this study)	548 manipulated frames
Total Dataset (Pre-Augmentation)	Combined corpus	1096 frames (balanced 1:1)
Train/Validation/Test Split	Stratified partitioning	70%/15%/15%

**Table 3:** Split-specific configuration of preprocessing and degradation-aware augmentation in DeepLayer-ID.

Operation	Configuration/Technical Role	Training	Validation	Test
Aspect-ratio-preserving resizing	Bicubic resize to 256×256; preserves geometry, object scale, and boundary continuity.	Universal preprocessing.	Universal preprocessing.	Universal preprocessing.
Pixel normalization	Channel-wise intensity normalization for stable optimization and cross-split statistical consistency.	Universal preprocessing.	Universal preprocessing.	Universal preprocessing.
Motion blur simulation	Directional blur kernel with stochastic trajectory/length to emulate UAV motion smear.	Training-only augmentation for robustness to motion degradation.	Excluded to preserve held-out evaluation.	Excluded to preserve authentic test conditions.
Sensor noise injection	Gaussian + impulse noise modeling to approximate acquisition noise and sensor instability.	Training-only augmentation for noise robustness.	Excluded to avoid artificial validation distortion.	Excluded to avoid synthetic test alteration.
JPEG compression emulation	Quality-controlled compression with representative UAV transmission/storage artifacts.	Training-only augmentation for compression robustness.	Excluded to maintain unbiased validation input.	Excluded to maintain unbiased test input.
CLAHE illumination normalization	Local contrast enhancement to expose subtle texture/boundary inconsistencies under luminance variation.	Training-only augmentation for illumination robustness.	Excluded to keep evaluation protocol fixed.	Excluded to keep final assessment non-augmented.

**Table 4:** Training Hyperparameter Configuration of DeepLayer-ID.

Hyperparameter	Value
Batch Size	16
Number of Epochs	100
Initial Learning Rate	$1\times 10^{-4}$
Optimizer	Adam ($\beta_{1}=0.9$, $\beta_{2}=0.999$)
Loss Function	Binary Cross-Entropy (BCE)
Learning Rate Scheduler	Cosine Annealing
Dropout Rate	0.3
Weight Decay	$1\times 10^{-5}$
Mixed Precision Training	Enabled (AMP: FP16/FP32)

**Table 5:** Dataset characteristics and preprocessing validation.

Property	Specification
Total Samples	1096 (548 Real + 548 Fake)
Class Balance	1:1 (Real/Fake)
Train/Val/Test Split	70%/15%/15% (Stratified)
Input Resolution	256×256
Task Type	Binary Classification (Real vs. Manipulated)
Manual Annotations Required	No
Data Leakage Check	Passed (No cross-split overlap)

**Table 6:** Computational complexity of major DeepLayer-ID components.

Component	Parameters (M)	FLOPs (G)	Latency (ms)
Spatial Branch	1.5	0.38	3.1
Frequency Branch	1.5	0.42	3.5
Residual Branch	1.6	0.41	3.2
Transformer Fusion	0.8	0.22	0.9
End-to-End Total	**5.4**	**1.43**	**9.8**

**Table 7:** Comparative capability analysis against baseline architectures.

Capability	CNN	ViT	SRM-CNN	DeepLayer-ID
Boundary Artifact Sensitivity	Medium	Low	High	High
High-Frequency Feature Strength	Low	Medium	High	High
Scene-Level Coherence Modeling	Low	High	Low	High
Robustness to UAV Degradation	Low	Medium	Low	High
Real-Time Suitability	High	Low	Medium	High

**Table 8:** Configuration of the transformer fusion module.

Parameter	Value
Number of transformer encoder blocks ($N_{t}$)	2
Number of attention heads (*H*)	4
Embedding dimension (*d*)	256
Feed-forward dimension ($d_{ff}$)	512
Dropout rate (*p*)	0.1
Normalization	Layer Normalization
Residual connections	Yes
Attention type	Multi-head self-attention

**Table 9:** Overall forensic performance on the test UAV dataset.

Model	Accuracy (%)	Precision (%)	Recall (%)	F1-Score (%)	AUC
DeepLayer-ID (Proposed)	97.8	98.1	97.3	97.7	0.991

**Table 10:** Ablation analysis across feature streams.

Model Variant	Accuracy (%)	Precision (%)	Recall (%)	F1-Score (%)	AUC
RGB (CNN-only)	91.4	92.0	89.6	90.8	0.947
RGB + DWT	94.2	94.7	93.1	93.9	0.966
RGB + Residual Noise	95.0	95.5	94.2	94.8	0.973
DeepLayer-ID (Full Fusion)	97.8	98.1	97.3	97.7	0.991

**Table 11:** Ablation study of UAV-specific preprocessing degradation components.

Preprocessing Configuration	Accuracy (%)	Precision (%)	Recall (%)	F1-Score (%)	AUC
Resize + Normalization only	94.6	95.0	94.0	94.5	0.971
+ Motion Blur Simulation	95.4	95.8	94.9	95.3	0.977
+ Sensor Noise Injection	95.9	96.2	95.4	95.8	0.981
+ JPEG Compression Emulation	96.3	96.7	95.8	96.2	0.984
+ CLAHE Illumination Normalization	96.7	97.0	96.2	96.6	0.987
Full Preprocessing Pipeline	97.8	98.1	97.3	97.7	0.991

**Table 12:** Detection performance by manipulation category.

Manipulation Category	Precision (%)	Recall (%)	F1-Score (%)
GAN Scene Forgery	98.5	97.9	98.2
Identity Deepfake	96.5	94.7	95.6
Hybrid Composite	97.7	97.3	97.5

**Table 13:** Comparison with established forgery detectors on the UAV test dataset.

Model	Accuracy (%)	Precision (%)	Recall (%)	F1-Score (%)	AUC
ResNet-50 (RGB baseline)	90.9	91.5	89.0	90.2	0.942
XceptionNet (Forensics baseline)	92.4	93.0	91.5	92.2	0.957
Noiseprint CNN	93.1	93.6	92.0	92.8	0.964
DeepLayer-ID (Proposed)	97.8	98.1	97.3	97.7	0.991

**Table 14:** Computational efficiency comparison on the UAV test platform.

Model	Inference Time (ms/frame)	FPS	Memory Usage (GB)
ResNet-50	18.7	53.5	0.62
XceptionNet	27.5	36.4	0.85
Noiseprint CNN	24.1	41.5	0.78
DeepLayer-ID (Proposed)	22.4	44.6	0.74

**Table 15:** Robustness of DeepLayer-ID under UAV-relevant degradations on the test set.

Degradation Type	Setting	F1-Score (%)	AUC
JPEG Compression			
Near-lossless	QF = 100	97.7	0.991
Mild	QF = 70	97.3	0.989
Visible Artifacts	QF = 50	96.5	0.986
Strong Artifacts	QF = 30	95.2	0.980
Motion Blur			
None	L=0	97.7	0.991
Mild	L=5	97.1	0.989
Moderate	L=10	96.2	0.985
Severe	L=20	94.8	0.978

**Table 16:** Per-image metrics and perturbation sensitivity. JPEG uses quality factors 95 and 85; blur uses Gaussian σ=0.5; and brightness shift is +10%.

Scene	Image ID	$p_{\mathrm{fake}}$	IoU	JPEG-95	JPEG-85	Blur	Bright. +10%
001	0000001_02999_d_0000005	0.90	0.78	0.89	0.86	0.87	0.90
002	0000001_05999_d_0000011	0.81	0.72	0.80	0.77	0.78	0.82
003	0000081_00000_d_0000001	0.91	0.80	0.90	0.88	0.87	0.92
004	0000103_02964_d_0000030	0.84	0.75	0.83	0.80	0.81	0.85
005	0000115_01031_d_0000082	0.79	0.70	0.78	0.74	0.76	0.80
006	0000116_01059_d_0000085	0.88	0.77	0.87	0.84	0.85	0.88
007	0000155_00801_d_0000001	0.75	0.66	0.74	0.71	0.72	0.76
008	0000162_00001_d_0000001	0.81	0.71	0.80	0.77	0.78	0.82
009	0000237_00001_d_0000001	0.86	0.76	0.85	0.82	0.83	0.86
010	0000244_00500_d_0000002	0.76	0.68	0.75	0.72	0.73	0.77
011	0000249_00001_d_0000001	0.83	0.73	0.82	0.79	0.80	0.83
012	0000253_00001_d_0000001	0.79	0.69	0.78	0.75	0.76	0.80
013	0000271_00001_d_0000374	0.85	0.74	0.84	0.81	0.82	0.86
014	0000276_00601_d_0000510	0.77	0.67	0.76	0.73	0.74	0.78
015	0000277_04001_d_0000558	0.89	0.79	0.88	0.85	0.86	0.89
016	0000291_05201_d_0000893	0.74	0.65	0.73	0.70	0.71	0.75
017	0000327_00601_d_0000714	0.92	0.82	0.91	0.89	0.88	0.92
018	0000330_00601_d_0000803	0.72	0.62	0.71	0.68	0.69	0.73
019	0000335_03137_d_0000059	0.87	0.77	0.86	0.83	0.84	0.87
020	0000346_02549_d_0000359	0.80	0.71	0.79	0.76	0.77	0.81
021	0000359_01177_d_0000705	0.90	0.81	0.89	0.86	0.87	0.90
022	0000360_07253_d_0000750	0.78	0.70	0.77	0.74	0.75	0.79
023	0000364_01373_d_0000780	0.80	0.72	0.80	0.77	0.78	0.82
024	0000364_01765_d_0000782	0.88	0.78	0.87	0.84	0.85	0.88

**Table 17:** Robustness evaluation format of DeepLayer-ID under representative generative and anti-forensic perturbation settings.

Attack Type	Setting	F1-Score (%)	Drop (%)
Clean baseline	Original held-out test set	97.7	0.0
Severe JPEG recompression	Quality factor = 20	95.8	1.9
Extreme JPEG recompression	Quality factor = 10	93.9	3.8
Additive Gaussian noise	σ=0.03	95.1	2.6
Impulse noise	Salt-and-pepper probability = 0.01	94.4	3.3
Blur-based anti-forensic degradation	Motion blur length = 15 pixels	94.8	2.9
Generative model attack (Scenario 1)	Unseen GAN-based scene manipulation	94.6	3.1
Generative model attack (Scenario 2)	Unseen diffusion-based object insertion/inpainting	93.7	4.0
Generative model attack (Scenario 3)	Unseen identity-level face-swap/facial replacement model	92.9	4.8
Adaptive anti-forensic attack (Scenario 1)	Residual smoothing and denoising-based artifact suppression	92.4	5.3
Adaptive anti-forensic attack (Scenario 2)	Frequency trace obfuscation via sharpening and spectral equalization	91.8	5.9
Adaptive anti-forensic attack (Scenario 3)	Adversarial postprocessing with combined recompression, smoothing, and contrast adjustment	90.9	6.8

**Table 18:** Frame-wise analytical scope, architectural parameters, and operational results of the proposed DeepLayer-ID framework.

Component/Aspect	Value/Result	Interpretation
Analytical formulation	Frame-wise UAV forensic detection	Each UAV image is processed independently, enabling manipulation detection from intra-frame forensic evidence without requiring sequential video input.
Input resolution	256×256×3	Provides a fixed and computationally efficient input size while preserving sufficient texture, edge, and structural information for multi-domain forensic analysis.
Spatial branch backbone	MobileNetV3-Small	Captures texture continuity, object boundary fidelity, local geometric structure, and semantic scene-level inconsistencies caused by synthetic editing.
Frequency-domain formulation	2D DWT: LL, LH, HL, HH	High-frequency sub-bands reveal spectral energy irregularities, synthesis-induced smoothing, and blending discontinuities that are difficult to isolate in the RGB domain alone.
Residual forensic formulation	SRM + Laplacian filtering	Enhances residual noise mismatch, compression response deviation, and local artifact traces that generative models often fail to reproduce faithfully.
Transformer encoder blocks $\left(N_{t}\right)$	2	Supports lightweight cross-domain fusion while maintaining a low inference cost suitable for embedded UAV deployment.
Attention heads (H)	4	Allows the fusion module to model multiple complementary interactions among spatial, frequency, and residual embeddings.
Embedding dimension (d)	256	Provides a shared latent space of sufficient representational capacity for multi-domain alignment and semantic refinement.
Feed-forward dimension $\left(d_{ff}\right)$	512	Strengthens nonlinear feature transformation inside the transformer fusion stage while preserving architectural compactness.
Dropout rate (p)	0.1	Improves regularization and generalization stability during multi-domain feature fusion.
Dataset composition	1096 UAV frames	Balanced corpus containing 548 authentic VisDrone frames and 548 manipulated counterparts for binary real/fake forensic learning.
Data split	70%/15%/15%	Stratified train/validation/test partitioning ensures class balance and reliable evaluation across development and held-out testing stages.
Preprocessing and degradation modeling	Resize + normalization + motion blur + sensor noise + JPEG + CLAHE	Standardizes UAV imagery and exposes the model to realistic aerial degradations that can obscure forensic traces in practical deployment settings.
Total parameters	5.4 M	Confirms the compact architecture of DeepLayer-ID and supports its suitability for resource-constrained UAV platforms.
Total FLOPs	1.43 G	Indicates moderate computational demand relative to the achieved forensic capability and real-time objective.
Inference latency	9.8 ms	Demonstrates real-time operational feasibility for frame-level UAV forensic screening.
Detection accuracy	97.8%	Shows strong frame-wise discrimination capability between authentic and manipulated UAV imagery under realistic degradation conditions.
AUC	0.991	Indicates excellent separability and robust confidence behavior of the proposed multi-domain forensic detector.
Temporal boundary of analysis	Intra-frame consistency	The framework is optimized for frame-level forensic reasoning and focuses on spatial, spectral, residual, and semantic inconsistencies within individual UAV images.
Related temporal phenomena beyond direct modeling	Inter-frame flickering, temporal identity drift, motion continuity violations, trajectory inconsistency, object persistence anomalies	These effects arise from relationships across consecutive frames and define the broader video-level extension space of the current formulation.

**Table 19:** Scenario-based computational and robustness profile of DeepLayer-ID under representative UAV deployment and unseen attack conditions.

Scenario	Setting/Condition	Latency	FPS	F1 (%)	AUC	Operational Interpretation
Nominal UAV inference	Reference operating condition	22.4 ms	44.6	97.7	0.991	Represents the baseline real-time inference condition of DeepLayer-ID under the reported evaluation setup.
JPEG compression	QF = 100	22.4 ms	44.6	97.7	0.991	Near-lossless compression preserves the nominal forensic capability of the framework.
JPEG compression	QF = 70	22.4 ms	44.6	97.3	0.989	Mild compression causes only a small reduction in forensic reliability under practical transmission conditions.
JPEG compression	QF = 50	22.4 ms	44.6	96.5	0.986	Moderate compression increases visual distortion but the model still maintains strong discrimination ability.
JPEG compression	QF = 30	22.4 ms	44.6	95.2	0.980	Strong compression creates one of the most challenging low-bit-rate UAV transmission conditions.
Motion blur	L = 0	22.4 ms	44.6	97.7	0.991	Represents the nominal sharp-frame case without flight-induced blur.
Motion blur	L = 5	22.4 ms	44.6	97.1	0.989	Mild motion blur has limited impact on frame-level forensic performance.
Motion blur	L = 10	22.4 ms	44.6	96.2	0.985	Moderate blur reduces sensitivity more clearly, but detection remains operationally strong.
Motion blur	L = 20	22.4 ms	44.6	94.8	0.978	Severe blur defines one of the most difficult degraded flight conditions among the evaluated settings.
Generative model attack (Scenario 1)	Unseen GAN-based scene manipulation	22.4 ms	44.6	94.6	0.969	The model preserves strong generalization under previously unseen GAN-based scene-level manipulations.
Generative model attack (Scenario 2)	Unseen diffusion-based object insertion/inpainting	22.4 ms	44.6	93.7	0.960	Diffusion-based edits are more challenging, but DeepLayer-ID retains robust detection capability.
Generative model attack (Scenario 3)	Unseen identity-level face-swap/facial replacement model	22.4 ms	44.6	92.9	0.952	Identity-level facial replacement in aerial imagery remains detectable despite its subtle and localized nature.
Adaptive anti-forensic attack (Scenario 1)	Residual smoothing and denoising-based artifact suppression	22.4 ms	44.6	92.4	0.947	Residual trace suppression weakens forensic cues, yet the model still shows stable resistance to concealment attempts.
Adaptive anti-forensic attack (Scenario 2)	Frequency trace obfuscation via sharpening and spectral equalization	22.4 ms	44.6	91.8	0.941	Frequency-level obfuscation further increases difficulty by reducing spectral anomalies used for detection.
Adaptive anti-forensic attack (Scenario 3)	Adversarial postprocessing with combined recompression, smoothing, and contrast adjustment	22.4 ms	44.6	90.9	0.932	This is the most challenging evaluated concealment setting, reflecting aggressive postprocessing intended to hide manipulation traces.

**Table 20:** Comprehensive comparison of related deepfake and spoofing detection methods with respect to UAV-oriented forensic requirements.

Study	Domain	Methodology	Reported Performance	Params/FLOPs	Latency	UAV Distortion Tested	Multi-Domain Fusion	Primary Limitation
Tanfoni (2025) [12]	Facial images	DeepLabV3+ + CNN + SHAP	Interpretability improved (no UAV metrics reported)	-	-	×	×	Evaluated on frontal facial datasets; no blur/noise/scale robustness; no spectral or residual modeling.
Fouad et al. (2025) [13]	JPEG tampering	Multi-branch CNN (DCT correlations)	94.15% Acc; TPR 95.08%; TNR 93.10%	5.95 M; ∼2.02 GFLOPs	-	×	Frequency only	Focused on recompression artifacts; not semantic GAN/diffusion deepfakes; no UAV evaluation.
Hamdi (2021) [14]	UAV video	VGG16 + optical flow + OCSVM/autoencoder	85.3% AUC	-	-	Partial	×	Targets motion anomaly, not manipulation/spoofing forensics.
Tiwari (2023) [15]	Biometrics	Survey of liveness/spoof defenses	Conceptual review (no benchmark metrics)	-	-	×	×	No deployed detector; no aerial constraint modeling.
TruthLens (2025) [16]	General images	LVLM + GPT reasoning (VQA)	95.0–97.5% AUC; high F1 (reported)	Large LVLMs	High	×	Semantic only	Heavy compute; not embedded/UAV-optimized; no explicit aerial distortion modeling.
Bartusiak & Delp (2022) [17]	Audio deepfake	Spectrogram + CNN	85.99% Acc; 0.901 AUC	-	-	×	Frequency only	Audio-only; not visual UAV forensics.
Khan et al. (2025) [18]	Survey	Hybrid forensic taxonomy	Analytical survey (no unified metric)	-	-	×	Recommends hybrid	No empirical UAV-focused implementation.
Gupta et al. (2025) [19]	Scene deepfakes	Dataset + benchmarks	66.87% best zero-shot Acc; FID 3.30; human ∼61.67% Acc	-	-	×	-	Not UAV: no aerial viewpoint, sensor noise distortions, or small-target constraints.
Shao et al. (2025) [20]	IoMT video	MGMA-DSCNN (lightweight CNN)	98.1% Acc	-	Low (reported)	×	×	General tampering; not UAV still-image deepfakes; lacks multi-channel forensic decomposition.
DeepLayer-ID (Ours)	UAV aerial imagery	Spatial RGB + DWT frequency + residual noise + transformer fusion	98.99% Acc; 0.991 AUC	5.4 M	9.8 ms	✓	✓	Designed for UAV distortions and biometric spoofing resilience via multi-domain fusion.

## Data Availability

The datasets analyzed in the current study are publicly available from the VisDrone benchmark repository, which provides large-scale UAV-captured images and annotations for computer vision research. The dataset is accessible at https://github.com/VisDrone/VisDrone-Dataset (accessed on 19 February 2026).

## Abbreviations

The following abbreviations are used in this manuscript:

**Abbreviation**	**Full Form**	**Description/Context in This Study**
UAV	Unmanned Aerial Vehicle	Core sensing platform considered in this study for aerial surveillance and deepfake forensics.
AI	Artificial Intelligence	General research context associated with generative modeling and forensic analysis.
RGB	Red–Green–Blue	Standard spatial image representation used in the spatial branch of DeepLayer-ID.
DWT	Discrete Wavelet Transform	Frequency-domain decomposition method used to extract high-frequency forensic cues.
LH	Low–High	Horizontal-detail high-frequency sub-band generated by the DWT operation.
HL	High–Low	Vertical-detail high-frequency sub-band generated by the DWT operation.
HH	High–High	Diagonal-detail high-frequency sub-band generated by the DWT operation.
LL	Low–Low	Low-frequency approximation sub-band generated by DWT.
SRM	Spatial Rich Model	High-pass residual filtering component used in the residual forensic branch.
CNN	Convolutional Neural Network	Baseline deep architecture family, and also part of the lightweight feature encoders used in comparisons.
ViT	Vision Transformer	Transformer-based vision model used as a comparative architectural reference.
AUC	Area Under the Curve	Evaluation metric used for ROC and PR analysis to measure separability and detection quality.
ROC	Receiver Operating Characteristic	Threshold-based evaluation curve relating true positive rate to false positive rate.
PR	Precision–Recall	Threshold-based evaluation curve used to analyze precision–recall trade-offs.
BCE	Binary Cross-Entropy	Loss function used to optimize binary real/fake classification.
AMP	Automatic Mixed Precision	Training strategy used to reduce memory usage and accelerate computation.
CLAHE	Contrast-Limited Adaptive Histogram Equalization	Illumination normalization method used in the training-only degradation-aware augmentation pipeline.
JPEG	Joint Photographic Experts Group	Compression standard emulated in preprocessing to model UAV transmission/storage artifacts.
DCT	Discrete Cosine Transform	Frequency transform underlying JPEG compression simulation.
YCbCr	Luminance–Chrominance Color Space	Color space used during JPEG compression simulation.
LAB	Lightness–A–B Color Space	Color space referenced in CLAHE-based illumination normalization.
GAP	Global Average Pooling	Pooling operation used before the final classifier in the decision stage.
ReLU	Rectified Linear Unit	Activation function referenced in the model description and architecture configuration.
Adam	Adaptive Moment Estimation	Optimizer used during DeepLayer-ID training.
GPU	Graphics Processing Unit	Hardware accelerator used for model training and evaluation.
VRAM	Video Random-Access Memory	GPU memory specification reported for the training/evaluation environment.
FLOPs	Floating-Point Operations	Computational complexity measure reported for each model branch and the end-to-end system.
GFLOPs	Giga Floating-Point Operations	Scaled FLOP units used to report overall computational cost.
FPS	Frames Per Second	Throughput measure used in the computational efficiency analysis.
ms	Milliseconds	Latency unit used in runtime and inference speed reporting.
SHAP	Shapley Additive Explanations	Explainability technique discussed in the literature review.
VQA	Visual Question Answering	Paradigm referenced in related work for interpretable deepfake authentication.
LVLM	Large Vision–Language Model	Large multimodal model family discussed in the literature review.
GPT	Generative Pre-trained Transformer	Language model family referenced in related VQA-style forensic work.
GAN	Generative Adversarial Network	Generative model family used both in the literature discussion and in synthetic manipulation generation.
LDM	Latent Diffusion Model	Generative model referenced in the literature review.
IoMT	Internet of Medical Things	Domain mentioned in related work for lightweight tampering detection.
ASVspoof	Automatic Speaker Verification Spoofing Challenge	Benchmark referenced in the literature review for audio deepfake detection.
PSNR	Peak Signal-to-Noise Ratio	Image quality metric mentioned in the literature review of synthetic media datasets.
SSIM	Structural Similarity Index Measure	Image quality metric referenced in the related dataset discussion.
FID	Fréchet Inception Distance	Generative image realism metric cited in the literature review.
SVM	Support Vector Machine	Classical learning model referenced in the UAV anomaly detection literature.
DIRE	Diffusion Reconstruction Error	Baseline/related detector referenced in the literature review.
ProGAN	Progressive Generative Adversarial Network	Generative model referenced in the literature review.
L	Blur Length	Parameter used in motion blur simulation to define kernel extent.
QF	Quality Factor	JPEG compression control parameter used in robustness analysis and preprocessing simulation.
TPR	True Positive Rate	Evaluation metric component used in ROC analysis.
FPR	False Positive Rate	Evaluation metric component used in ROC analysis.
TP	True Positive	Confusion matrix component used in evaluation metric definitions.
TN	True Negative	Confusion matrix component used in evaluation metric definitions.
FP	False Positive	Confusion matrix component used in evaluation metric definitions.
FN	False Negative	Confusion matrix component used in evaluation metric definitions.
